# Non-blinking (Zn)CuInS/ZnS Quantum Dots Prepared by *In Situ* Interfacial Alloying Approach

**DOI:** 10.1038/srep15227

**Published:** 2015-10-13

**Authors:** Aidi Zhang, Chaoqing Dong, Liang Li, Jinjin Yin, Heng Liu, Xiangyi Huang, Jicun Ren

**Affiliations:** 1 School of Chemistry and Chemical Engineering, State Key Laboratory of Metal Matrix Composites, Shanghai Jiao Tong University, 800 Dongchuan Road, Shanghai 200240, China; 2 School of Environmental Science and Engineering, Shanghai Jiao Tong University, 800 Dongchuan Road, Shanghai 200240, China

## Abstract

Semiconductor quantum dots (QDs) are very important optical nanomaterials with a wide range of potential applications. However, blinking behavior of single QD is an intrinsic drawback for some biological and photoelectric applications based on single-particle emission. Herein we present a rational strategy for fabrication of non-blinking (Zn)CuInS/ZnS QDs in organic phase through *in situ* interfacial alloying approach. This new strategy includes three steps: synthesis of CuInS QDs, eliminating the interior traps of QDs by forming graded (Zn)CuInS alloyed QDs, modifying the surface traps of QDs by introducing ZnS shells onto (Zn)CuInS QDs using alkylthiols as sulfur source and surface ligands. The suppressed blinking mechanism was mainly attributed to modifying QDs traps from interior to exterior via a step-by-step modification. Non-blinking QDs show high quantum yield, symmetric emission spectra and excellent crystallinity, and will enable applications from biology to optoelectronics that were previously hindered by blinking behavior of traditional QDs.

Colloidal semiconductor quantum dots (QDs) are very important optical nanomaterials with a wide range of potential applications due to their excellent physical and chemical properties^1^,^2^,^3^,^4^. However, QDs, at the single-particle level, show severe fluorescence intermittency (or blinking) on a wide time scale from milliseconds to minutes, switching randomly between bright (on) and dark (off) states. This blinking behavior of QDs is an intrinsic drawback for certain biological and photoelectric applications that rely on single-particle emission, such as single nanoparticle tracking^5^,^6^ and single-photon light sources^7^,^8^. The blinking is generally considered to be from the charging-discharging process of individual QDs, in which an electron (or a hole) is temporarily lost to the surrounding matrix through the Auger recombination or captured by surface traps^9^. How to suppress or eliminate the QDs blinking has been becoming very important issues in the basic research of QDs^9^,^10^,^11^,^12^,^13^,^14^,^15^ since the blinking behavior was first reported in 1996^16^. So far, certain approaches are used to suppress the blinking behavior of QDs, which mainly includes the modification of small molecular ligands and introduction of inorganic shell (CdS and ZnS) on QDs surfaces to eliminate the QDs surface traps. Ha’s group observed that the QDs blinking was near-completely suppressed in certain thiol compound aqueous solution, which was probably attributed to the modification of QDs surface traps with thiol compound^17^. Our group found that the blinking behavior of CdTeS QDs were able to be controlled by synthetic conditions such as the structure and concentration of thiol compounds^18^,^19^. Under the optimal condition, we prepared non-blinking CdTeS alloyed QDs in aqueous solution by using thiol compounds as sulfur source and surface ligands. Furthermore, this similar operation was also used to significantly improve the “on time” fraction of InP/ZnS QDs^20^ and CdSe/CdS QDs^21^ prepared in organic phase by using alkylthiols as surface ligands and sulfur source. Klimov’s group and Dubertret’s group reported that the blinking behavior and photobleaching of QDs were dramatically suppressed by the introduction of a thick CdS shell to CdSe QDs, and the sizes of these “giant” QDs were significantly increased to 13–20 nm in this case^13^,^22^. Krauss’s group found that the QDs blinking was completely suppressed by formation of the graded interface between materials comprising QDs. Interestingly, as-prepared CdZnSe/ZnSe QDs possessed broad luminescent multiple emission peaks^10^. Bawendi’s group dramatically suppressed the blinking behavior of CdSe/CdS QDs by using octanethiol as sulfur source at high synthesis temperature, and the “on time” fraction of QDs were enhanced to 94%^12^. Recently, Peng’s group prepared phase-pure zinc-blende CdSe/CdS core/shell QDs with non-blinking behavior (“on time” fraction ≥ 95%)^23^. Although great progress have been made in suppressing QDs blinking, to prepare non-blinking QDs (“on time” fraction = 100%) with high quantum yield, symmetric emission spectrum and excellent crystallinity is still a great challenge. On the other hand, the current studies mostly focused on the blinking behavior of CdSe and CdTe QDs^9^,^11^,^12^,^13^,^14^,^16^,^17^,^18^,^19^,^22^,^23^,^24^,^25^. Up to now, it is not clear whether CuInS QDs possess blinking behavior. Compared with CdSe and CdTe QDs, CuInS QDs consist of non-toxic elements, and possess good biocompatibility and optical properties, and will become a promising candidate probes for biological applications^26^,^27^,^28^,^29^,^30^.

Herein, we present a new strategy for fabrication of non-blinking (Zn)CuInS/ZnS QDs in organic phase by *in situ* interfacial alloying approach. This strategy includes three steps, synthesis of CuInS QDs, eliminating the interior traps of QDs by forming graded (Zn)CuInS alloyed QDs, modifying the surface traps of QDs by introduction of ZnS shells onto (Zn)CuInS alloyed QDs. We investigated the effects of some reaction conditions on the QDs blinking, and found that the stoichiometric ratios of cations played a critical role in the fabrication of non-blinking QDs. Furthermore, we investigated the reaction mechanism and found that the suppression of QDs blinking was mainly attributed to the cation exchange process for the formation of graded (Zn)CuInS alloyed QDs. In this process, original cations (Cu^1+^ and In^3+^) diffuse out of the lattice under low reaction temperature, and then the free cation (Zn^2+^) rapidly occupy the certain vacancies of (Zn)CuInS alloyed QDs, which result in the suppression of QDs blinking. In the optimized condition, the as-prepared QDs exhibit non-blinking, high quantum yield, narrow and symmetric emission spectrum and excellent crystallinity.

## Results

### Single-particle and ensemble optical properties of the (Zn)CuInS alloyed QDs

**Figure 1:**
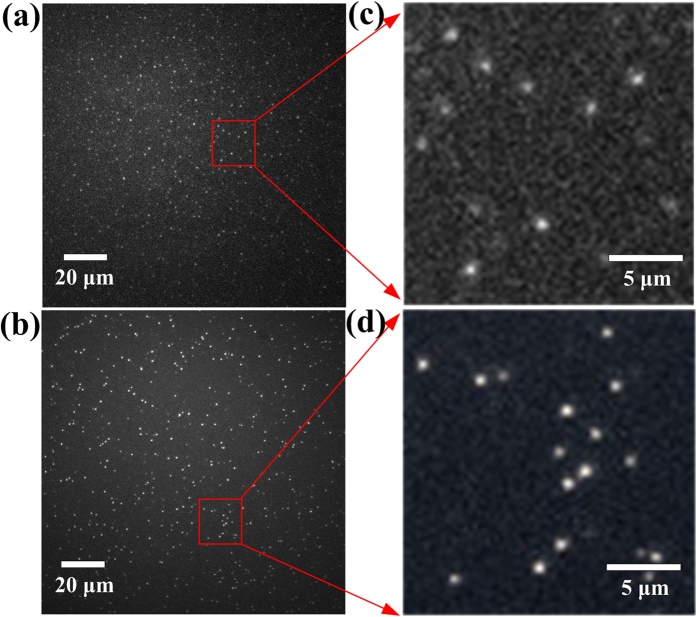
TIRFM images of CuInS-based QDs. (**a**) Whole frame of TIRFM images of (Zn)CuInS alloyed QDs (Cu:In:Zn stoichiometric ratio of 1:4:3) dispersed with organic solvent (90% hexane-10% octane) on glass substrate at room temperature (512 × 512 pixel). (**b**) Magnification of the selected area from (**a**). (**c**) Whole frame of TIRFM image of (Zn)CuInS/ZnS QDs (Cu:In:Zn stoichiometric ratio of 1:4:3, the growth time of ZnS shell is 10 h) dispersed with organic solvent (90% hexane-10% octane) on glass substrate at room temperature (512 × 512 pixel). (**d**) Magnification of the selected area from (**c**). All the QDs are well separated and no aggregation is observed in this case. The exposure time is 50 ms.

In this study, single QD fluorescence experiments have been performed with a total internal reflection fluorescence microscopy (TIRFM) imaging system as described in the supplementary information (Supplementary Fig. S1). To record the fluorescence time trace of a single particle, the intensity of an isolated fluorescent feature in the image is recorded as a function of time under constant illumination with a 488 nm continuous argon ion laser. Figure 1a,b show two typical whole frame fluorescence images of isolated (Zn)CuInS alloyed QDs and (Zn)CuInS/ZnS core/shell QDs, Fig. 1c,d show a magnification of the selected area of several (Zn)CuInS and (Zn)CuInS/ZnS QDs. As shown in these images, individual QDs are uniformly dispersed, and no aggregation is observed in these cases. These results demonstrate that our single-particle fluorescent imaging system has high sensitivity, and is a very powerful detection tool to simultaneously monitor the single-particle fluorescent emission behavior of several hundred or up to thousands QDs. Meanwhile, this result also illustrated the preparation of QDs samples was successful for single particle imaging. Furthermore, some experimental procedures were performed to confirm that all measurements were associated with a single dot (see Supplementary Discussion for more details).

**Figure 2:**
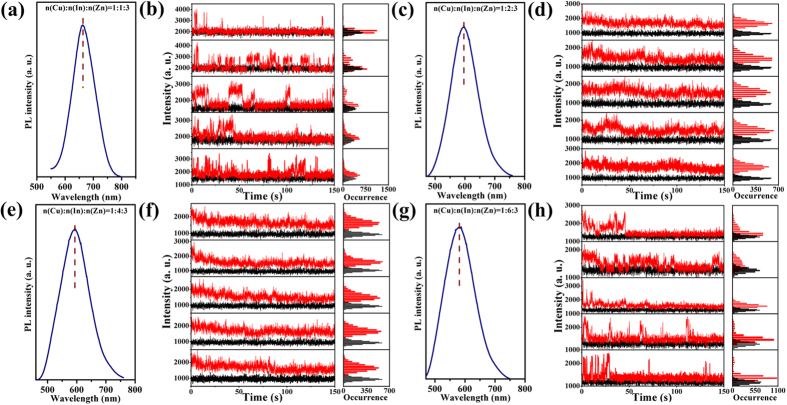
Ensemble and single-particle optical properties of (Zn)CuInS alloyed QDs. (**a**,**c**,**e**,**g**) PL spectra for (Zn)CuInS alloyed QDs with different stoichiometric ratios of Cu:In:Zn (1:1:3, 1:2:3, 1:4:3, and 1:6:3). The PL curves were obtained by exciting the samples at 450 nm. (**b**,**d**,**f**,**h**) Temporal evolution of representative fluorescence-intensity trajectories for individual (Zn)CuInS alloyed QDs with different stoichiometric ratios of Cu:In:Zn (1:1:3, 1:2:3, 1:4:3, and 1:6:3). Histograms to right indicate the corresponding distribution of intensities observed in the trajectories. All QDs were excited by continuous 488 nm Argon ion laser. The data were recorded by an EMCCD with offset correction. The binning time is 50 ms. The horizontal black line is the intensity of the background fluorescence signal.

We investigated the effects of alloyed QDs formation on single-particle optical properties. Four different (Zn)CuInS alloyed QDs (Cu:In:Zn stoichiometric ratios of 1:1:3, 1:2:3, 1:4:3, and 1:6:3) were synthesized in organic phase (see the Method section). It should be pointed out that the photoluminescence (PL) of original CuInS QDs was very weak (Supplementary Table S1), and we could only obtain very weak PL signal by the TIRFM imaging system because the signal to noise ratio of CuInS QDs was poor. It was very difficult to distinguish the “on” and “off” events using a uniform data processing procedure (see Methods section, single-dot PL signal collection, process and analysis). However, the fluorescent intensity of (Zn)CuInS alloyed QDs was dramatically increased by the surface etching of CuInS QDs with Zn^2+^ ion (Supplementary Table S2). The fluorescence-intensity trajectories of single-particle and ensemble emission spectra of (Zn)CuInS alloyed QDs are shown in Fig. 2. Single-particle trajectories from individual QDs were obtained with a bin time of 50 ms. For clearly distinguishing the “on” and “off” events, the threshold was set to three times the standard deviation above the average background intensity. The fluorescence-intensity trajectories shown in Fig. 2d,f demonstrate that these alloyed QDs (Cu:In:Zn stoichiometric ratios of 1:2:3 and 1:4:3) exhibit almost non-blinking behavior (Supplementary Video S1). However, the other two alloyed QDs (Cu:In:Zn stoichiometric ratios of 1:1:3 and 1:6:3) display severe blinking behavior, as shown in Fig. 2b,h (Supplementary Video S2). The random switching of their PL intensity between the “on time” and “off time” periods can be clearly resolved. The percentages distribution of the statistical QDs for different “on time” fractions were processed with homemade programs (more than 100 dots are randomly selected and measured). Briefly, on-time % represents the “on time” fractions of QDs in the total observation time of 3 min. A “non-blinking” QD is defined when the “on time” fraction exceeds 99% of the whole observation time. For the alloyed QDs with Cu:In:Zn stoichiometric ratios of 1:2:3 and 1:4:3, their percentages of non-blinking QDs were about 89.2% and 87.1%, respectively (Supplementary Fig. S2). While the percentages of non-blinking QDs for Cu:In:Zn stoichiometric ratios of 1:1:3 and 1:6:3 were 5.32% and 5.12%, respectively (Supplementary Fig. S2). Statistical analysis documented that both the “on time” and “off time” events (*m*_on_ and *m*_off_) followed the power-law distribution 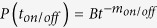
13,20,31. The fitting results (more than 100 dots are randomly selected and measured) of the power-law exponents (*m*_on_ or *m*_off_) express the statistics of “on time” and “off time” (Supplementary Figs S3 and S4). The *m*_on_ exponents for blinking (Zn)CuInS alloyed QDs (Cu:In:Zn stoichiometric ratios of 1:1:3 and 1:6:3) were 1.39 and 1.53. The *m*_off_ exponents were 1.49 and 1.55. The results above illustrate that the stoichiometric ratios of cations (Cu, In, and Zn) was a very important factor affecting the single-particle fluorescent emission behavior of the (Zn)CuInS alloyed QDs.

The ensemble PL properties of (Zn)CuInS alloyed QDs are demonstrated in Fig. 2(a,c,e,g), and their PL emission peaks are 675, 596, 595, and 580 nm, respectively. The PL peaks of (Zn)CuInS alloyed QDs exhibit a blue shift of about 35 nm compared with the original CuInS QDs (Supplementary Table S1 and S2), which was due to surface etching of CuInS QDs^29^,^32^,^33^,^34^ and the indium content variation in the (Zn)CuInS alloyed QDs^35^,^36^,^37^. The PL QYs of these alloyed QDs samples were about 3.8%, 16.8%, 24.9%, and 36.3%, respectively (Supplementary Fig. S5). This result documented that surface etching of CuInS QDs with Zn^2+^ ion dramatically enhanced the PL QYs of (Zn)CuInS QDs^29^,^38^.

### Single-particle and ensemble optical properties of the (Zn)CuInS/ZnS core/shell QDs

**Figure 3:**
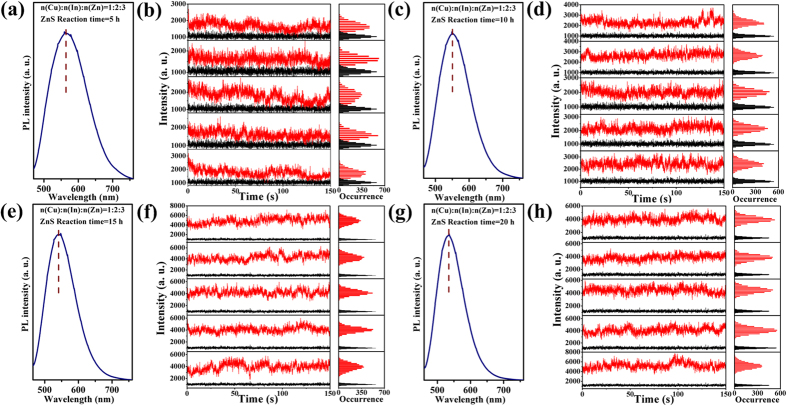
Ensemble and single-particle optical properties of (Zn)CuInS/ZnS QDs (Cu:In:Zn stoichiometric ratio of 1:2:3). (**a**,**c**,**e**,**g**) PL spectra for (Zn)CuInS/ZnS QDs with different ZnS shell growth time (5, 10, 15, and 20 h, respectively). The PL curves were obtained by exciting the samples at 450 nm. (**b**,**d**,**f**,**h**) Temporal evolution of representative fluorescence-intensity trajectories for individual (Zn)CuInS/ZnS QDs with different ZnS shell growth time (5, 10, 15, and 20 h, respectively). Histograms to right indicate the corresponding distribution of intensities observed in the trajectories. All QDs were excited by continuous 488 nm Argon ion laser. The data were recorded by an EMCCD with offset correction. The binning time is 50 ms. The horizontal black line is the intensity of the background fluorescence signal.

We investigated the effects of ZnS shell formation on single-particle optical properties of QDs. In this study, four different (Zn)CuInS alloyed QDs (Cu:In:Zn stoichiometric ratios of 1:1:3, 1:2:3, 1:4:3, and 1:6:3) were used to prepare the (Zn)CuInS/ZnS core/shell QDs, and the procedures were described in the Method section. These (Zn)CuInS/ZnS QDs show different single-particle optical properties although they were under the same ZnS shell growth conditions. Figure 3 shows the temporal evolution of single-particle optical properties for (Zn)CuInS/ZnS QDs (Cu:In:Zn stoichiometric ratio of 1:2:3). These QDs exhibit non-blinking behavior during the growth process of 20 h (Supplementary Video S3,S4). The statistical percentages of non-blinking QDs for different growth stages were 94.7% (5 h), 97.0% (10 h), 98.4% (15 h), and 98.0% (20 h), respectively (Supplementary Fig. S6).

**Figure 4:**
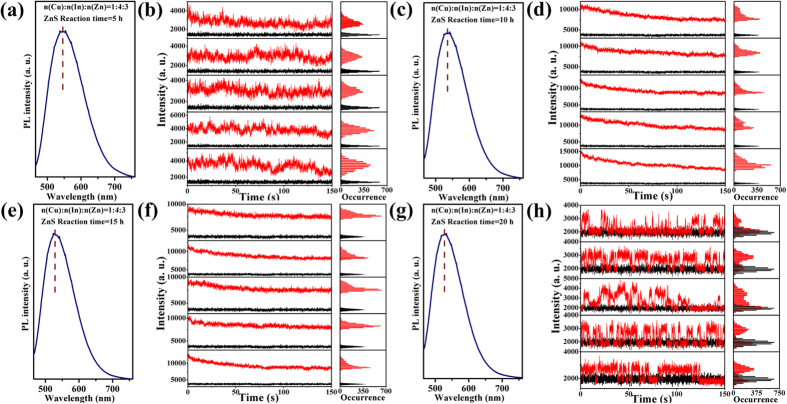
Ensemble and single-particle optical properties of (Zn)CuInS/ZnS QDs (Cu:In:Zn stoichiometric ratio of 1:4:3). (**a**,**c**,**e**,**g**) PL spectra for (Zn)CuInS/ZnS QDs with different ZnS shell growth time (5, 10, 15, and 20 h, respectively). The PL curves were obtained by exciting the samples at 450 nm. (**b**,**d**,**f**,**h**) Temporal evolution of representative fluorescence-intensity trajectories for individual (Zn)CuInS/ZnS QDs with different ZnS shell growth time (5, 10, 15, and 20 h, respectively). Histograms to right indicate the corresponding distribution of intensities observed in the trajectories. All QDs were excited by continuous 488 nm Argon ion laser. The data were recorded by an EMCCD with offset correction. The binning time is 50 ms. The horizontal black line is the intensity of the background fluorescence signal.

Figure 4 demonstrates the ensemble PL spectra and temporal evolution of single-particle optical properties for (Zn)CuInS/ZnS QDs (Cu:In:Zn stoichiometric ratio of 1:4:3). These QDs show interesting single-particle optical properties. Within 15 h of shell growth, these QDs also display non-blinking behavior, and the statistical percentages of non-blinking QDs for different growth stages were 90.2% (5 h), 94.5% (10 h), and 98.6% (15 h), respectively (Supplementary Fig. S7). However, a transition from non-blinking to blinking was observed over the reaction time of 20 h (Supplementary Video S5). In this case, the statistical percentage of non-blinking QDs was 42.1% (20 h, Supplementary Fig. S7). Figure S8 shows the “on time” and “off time” events probability of the (Zn)CuInS/ZnS QDs (Cu:In:Zn stoichiometric ratio of 1:4:3, 20 h). The exponents of *m*_on_ and *m*_off_ for (Zn)CuInS/ZnS QDs (Cu:In:Zn stoichiometric ratio of 1:4:3) were 1.25 and 2.06. Although the core/shell structure can greatly reduce the lattice stress by the epitaxial growth on the core surface, there is still much accumulated stress in the core/shell boundary caused by the lattice mismatch during the fast epitaxial growth of the shell, which possibly triggers the interfacial defects^39^.

We also investigated the effects of ZnS shell formation on single-particle optical properties of the other two (Zn)CuInS/ZnS QDs (Cu:In:Zn stoichiometric ratios of 1:1:3 and 1:6:3). The (Zn)CuInS alloyed QDs possessed blinking behavior as described above. Interestingly, these core/shell QDs still show blinking behavior during the shell growth of 20 h. It should be noted that, the QDs blinking are significantly suppressed and higher “on time” fractions of QDs can be obtained after the introduction of ZnS shell (Supplementary Figs S9 and S10). However, we could not obtain non-blinking (Zn)CuInS/ZnS QDs by regulating the thickness of ZnS shell in this case. The results above demonstrate that the shell growth of QDs significantly affect the blinking behavior of QDs.

The ensemble PL properties of (Zn)CuInS/ZnS QDs (Cu:In:Zn stoichiometric ratios of 1:2:3 and 1:4:3) are demonstrated in Figs 3(a,c,e,g) and 4(a,c,e,g). The PL spectra show three obvious characteristics. First, the PL emission peaks of all the (Zn)CuInS/ZnS QDs gradually shifted toward shorter wavelengths than the (Zn)CuInS alloyed QDs with the introduction of ZnS shell, and reached balance at about 530–540 nm (Supplementary Fig. S11). This continuous blue shift of PL spectra was originated from the gradual Zn diffusion into the inner core of the nanocrystals by cation exchange process at elevated temperature^27^,^38^,^40^ (see the Discussion about elemental composition analysis), as well as compressive lattice strain applied to (Zn)CuInS alloyed QDs by the formation of inorganic shell layer with smaller lattice parameters^32^,^41^. Second, the full-width-at-half-maximum (FWHM) of the (Zn)CuInS/ZnS QDs became larger than the (Zn)CuInS alloyed QDs after the first 5 h shell growth, and then reduced in the following stages (10, 15, and 20 h) due to the improvement in size and shape uniformity of the nanocrystals. Third, the symmetry of PL spectra became poor with the injection of shell precursors. This phenomenon indicated the variation of bandgap energy, which may be caused by the cation exchange process of the nanocrystals.

During the shell growth, the PL QYs of the (Zn)CuInS/ZnS QDs (Cu:In:Zn stoichiometric ratio of 1:2:3) gradually increased, and then reached the highest QYs of 74% at the growth time of 9 h (Supplementary Fig. S12). Figure S13 demonstrates the QYs evolution of the (Zn)CuInS/ZnS QDs (Cu:In:Zn stoichiometric ratio of 1:4:3). At first stage, the QYs rapidly reached about 57% with growth time of 5 h, and then maintained about 50–60% in the following 10 h. The QY reached the highest value of 73% with growth time of 16 h, and then slightly decreased with further shell growth. The generated defects in the ZnS shell may be the source of new nonradiative recombination sites, which causes the decline of PL emission quality with thicker ZnS shell. Similarly, the QYs variations were found in other (Zn)CuInS/ZnS QDs with different stoichiometric ratios of Cu:In:Zn.

### Structural and compositional properties of the CuInS, (Zn)CuInS, (Zn)CuInS/ZnS QDs

**Figure 5:**
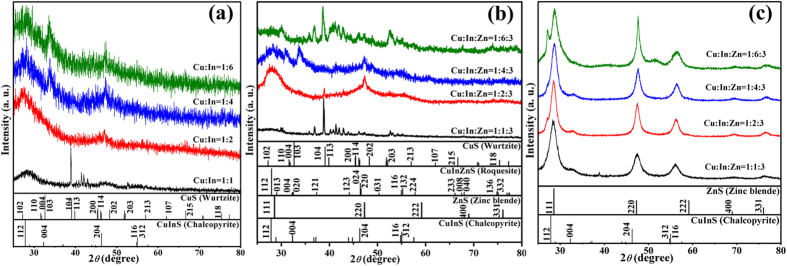
XRD patterns. (**a**) XRD patterns of CuInS QDs synthesized with different stoichiometric ratios of Cu:In (1:1, 1:2, 1:4 and 1:6). (**b**) XRD patterns of the (Zn)CuInS QDs synthesized with different stoichiometric ratios of Cu:In:Zn (1:1:3, 1:2:3, 1:4:3, and 1:6:3). (**c**) XRD patterns of the (Zn)CuInS/ZnS QDs synthesized with different stoichiometric ratios of Cu:In:Zn (1:1:3, 1:2:3, 1:4:3, and 1:6:3). The vertical bars correspond to standard data for CuInS (vertical bars, JCPDS card No. 32-0339), ZnS (vertical bars, JCPDS card No. 02-0564), CuInZnS (vertical bars, JCPDS card No. 47-1371), and CuS (vertical bars, JCPDS card No. 17-0449).

The X-ray diffraction patterns of CuInS QDs are shown in Fig. 5a. For CuInS QDs (Cu:In stoichiometric ratios of 1:2, 1:4, and 1:6), there are an intense peak at around 2*θ* = 27.8^o^, oriented along the (112) direction and other prominent peaks observed at around 47.0^o^ (204) and 54.8^o^ ((312)/(116)), which are signatures of the chalcopyrite structure (JCPDS file 32-0339). However, the XRD patterns of CuInS QDs (Cu:In stoichiometric ratio of 1:1) shows poor crystal structure and exhibits the coexistence of characteristic peaks of both CuInS chalcopyrite structure (JCPDS file 32-0339) and CuS wurtzite structure (JCPDS file 17-0449). After the ion exchange process, the characteristic peak positions of the (112), (204) and (312) crystal faces in the (Zn)CuInS alloyed QDs (Cu:In:Zn stoichiometric ratios of 1:2:3 and 1:4:3) show a shift to higher 2*θ* with respect to the CuInS phase. The lattice fringes in the HR-TEM images (Supplementary Fig. S20) are compatible with typical lattice sets (112) and (220) of chalcopyrite-like structure, showing *d*-spacings and vector relationships consistent with the quaternary roquesite CuInZnS system (JCPDS file 47-1371) (Fig. 5b). Because the broad XRD patterns and the lattice sets observed under HR-TEM can be compatible with a wide set of materials, neither XRD nor HR-TEM can be conclusive on the actual phase composition of these alloyed QDs^28^,^38^,^42^,^43^,^44^. Moreover XRD phase identification analysis documented that the (112) crystal face disappeared and there existed some Cu_x_S_y_ species (JCPDS file 17-0449) in the alloyed QDs (Cu:In:Zn stoichiometric ratios of 1:1:3 and 1:6:3). This data meant that the basic chalcopyrite-like structure of CuInS phase was destroyed in these two alloyed QDs. The results in Fig. 5b also illustrated that the initial stoichiometric ratios of cations in CuInS QDs greatly affected their crystal structure evolution in the ion exchange process. After the formation of ZnS shell, the diffraction peaks of all QDs shifted obviously towards the characteristic positions of cubic ZnS (Fig. 5c), and all samples show zinc blende structure (JCPDS file 02-0564), implying the formation of the (Zn)CuInS/ZnS core/shell structure.

Figure S14 and S15 show representative TEM images of CuInS QDs with Cu:In ratios of 1:2 and 1:4, and their average sizes are about 2.1 and 2.2 nm. Their sizes are widely distributed in the range of 2.0–2.5 nm (here, the size is defined as the height of the pyramid base measured by TEM, Supplementary Figs S16 and S17). The distance (0.31 nm) between the adjacent lattice fringes are the interplanar distances of CuInS (112) plane, agreeing well with the (112) *d* spacing of the literature value of 0.319 nm (JCPDS File No. 32-0339)^45^. The CuInS QDs (Cu:In ratios of 1:1 and 1:6) possessed different growth rate and showed wider size distributions (from 2.0 nm to 5.0 nm) under the same growth condition (Supplementary Figs S18 and S19). The different sizes distribution indicated that the stoichiometric ratios of cations greatly influenced the growth rate of the nanocrystals.

Significant improvements in the uniformity of size and shape of the (Zn)CuInS alloyed QDs were obtained after zinc stearate etching, as confirmed by the TEM images. These alloyed QDs displayed a near pyramidal shape. The detailed analysis indicates that these QDs are nearly monodisperse, exhibiting average sizes of about 1.7 and 1.9 nm for Cu:In:Zn stoichiometric ratios of 1:2:3 and 1:4:3 (Supplementary Fig. S20–S23). Continuous lattice fringes were clearly observed, indicating the high crystallinity of these QDs with the interplanar distance of 0.318 nm (Supplementary Fig. S20). Similarly, wide size distributions were also found in other (Zn)CuInS alloyed QDs (Cu:In:Zn ratios of 1:1:3 and 1:6:3, Supplementary Figs S24 and S25).

**Figure 6:**
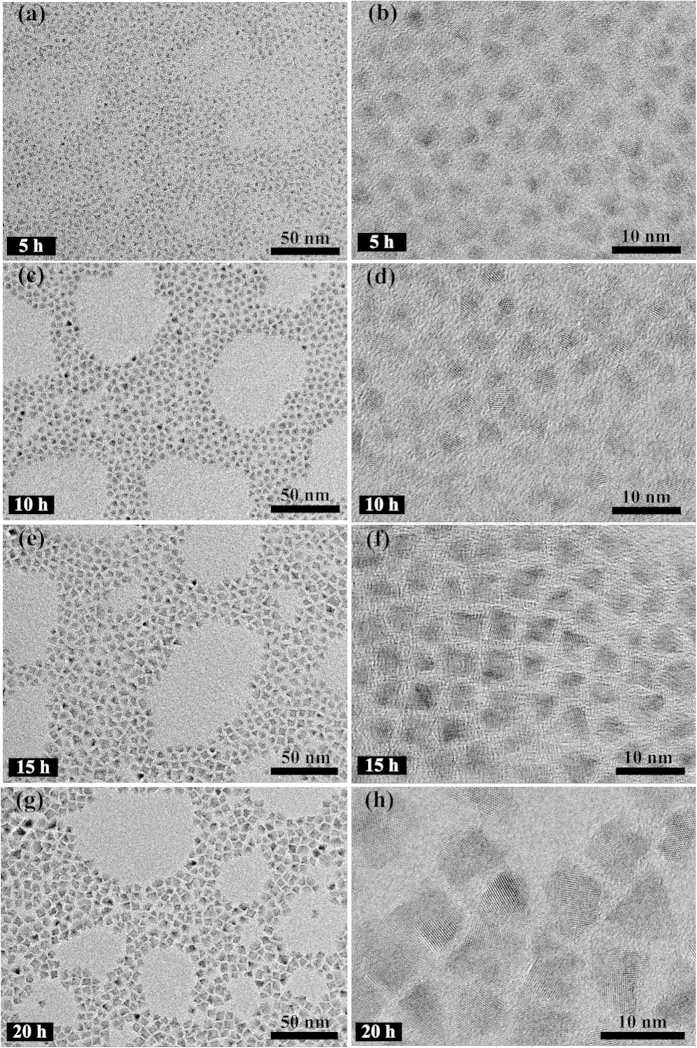
Representative TEM images of (Zn)CuInS/ZnS QDs (Cu:In:Zn stoichiometric ratio of 1:2:3) with different ZnS shell growth time (5, 10, 15, and 20 h, respectively). (**a**,**b**) 5 h, (**c**,**d**) 10 h, (**e**,**f**) 15 h, (**g**,**h**) 20 h.

**Figure 7:**
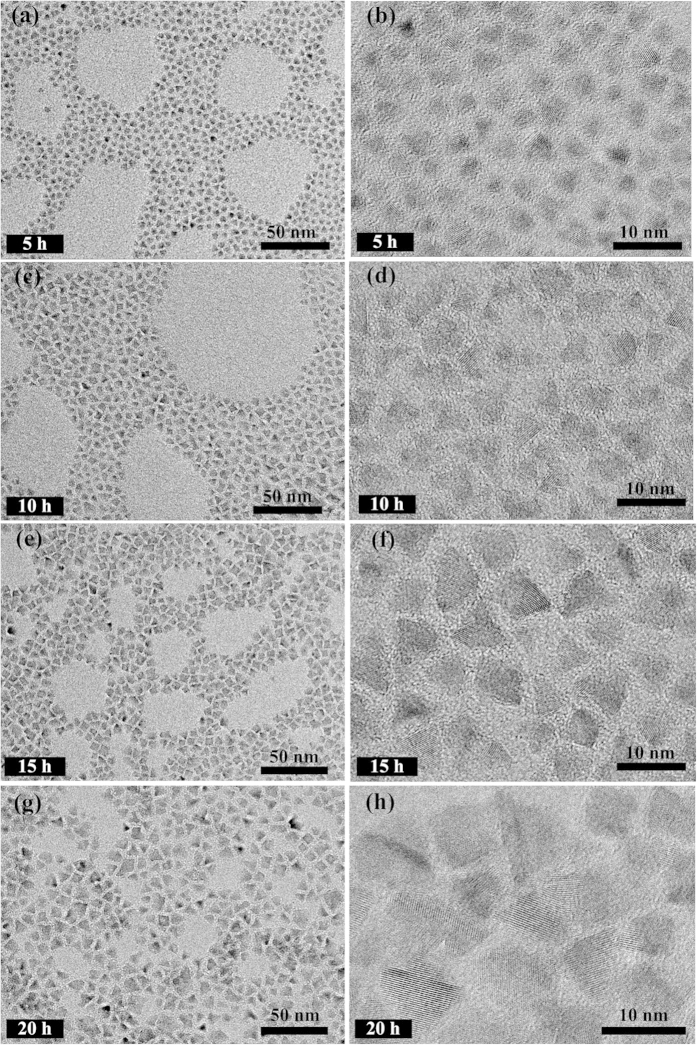
Representative TEM images of (Zn)CuInS/ZnS QDs (Cu:In:Zn stoichiometric ratio of 1:4:3) with different ZnS shell growth time (5, 10, 15, and 20 h, respectively). (**a**,**b**) 5 h, (**c**,**d**) 10 h, (**e**,**f**) 15 h, (**g**,**h**) 20 h.

Figure 6 shows the representative TEM images of (Zn)CuInS/ZnS QDs (Cu:In:Zn stoichiometric ratio of 1:2:3). The QDs sizes gradually increase with continuous introduction of shell precursors, which indicates the formation of core/shell structure. The TEM images of (Zn)CuInS/ZnS QDs also revealed the pyramidal shape and good monodispersity. The average sizes of these QDs are 3.0 nm (5 h), 4.6 nm (10 h), 5.6 nm (15 h), and 6.9 nm (20 h), respectively. Figure 7 shows the TEM images of the (Zn)CuInS/ZnS QDs (Cu:In:Zn stoichiometric ratio of 1:4:3). The average sizes of these QDs are 3.7 nm (5 h), 6.0 nm (10 h), 7.4 nm (15 h), and 8.3 nm (20 h), respectively. The size variations indicated that the stoichiometric ratios of cations had significant influence on the growth rate of ZnS shell. The higher growth rate resulted in bigger QDs with non-spherical shapes^26^,^28^. More detailed information about TEM images of (Zn)CuInS/ZnS QDs (Cu:In:Zn stoichiometric ratios of 1:1:3 and 1:6:3) were given in the supplementary information (Supplementary Fig. S26–S29).

Variations in optical properties correlate well with elemental composition, as determined via inductively coupled plasma optical emission spectrometer (ICP-OES) and energy-dispersive spectroscopy (EDS). The actual molar ratios of Cu:In in the CuInS QDs (Cu:In stoichiometric ratios of 1:1, 1:2, 1:4, and 1:8) are about 1.0:1.1, 1.0:1.2, 1.0:2.0, and 1.0:2.5, respectively (Supplementary Table S1). The actual cationic compositions are significantly different from the stoichiometric ratios of starting precursors. After ion exchange process, the actual molar ratios of (Zn)CuInS alloyed QDs (Cu:In:Zn stoichiometric ratios of 1:1:3, 1:2:3, 1:4:3, and 1:8:3) are about 1.0:4.6:1.6, 1.0:4.0:2.3, 1.0:4.5:1.9, and 1.0:3.4:1.6, respectively (Supplementary Table S2).

For (Zn)CuInS/ZnS QDs (Cu:In:Zn initial stoichiometric ratio of 1:2:3), the actual molar ratios of Cu:In:Zn in the shell growth process (5, 10, 15, 20 h) are 1.0:1.4:31.0, 1.0:1.3:33.1, 1.0:1.3:34.8, and 1.0:1.4:37.0, respectively (Supplementary Table S3). The actual molar ratios of Cu:In maintained a near constant in the whole growth process, which indicated that the cationic exchange process of Zn^2+^ with In^3+^ and Cu^1+^ was completed or reached the equilibrium before shell growth. Meanwhile, the relatively slow decrease in the molar ratios of Cu:Zn demonstrated the gradual formation process of core/shell structure. Interestingly, for In-rich (Zn)CuInS/ZnS QDs (Cu:In:Zn initial stoichiometric ratio of 1:4:3), the actual molar ratios of Cu:In maintained a relatively slow increase in the whole shell growth stages (1.0:2.9, 1.0:2.4, 1.0:2.0, and 1.0:1.1) (Supplementary Table S4). The continuous decrease of In^3+^ content means that cationic exchange process still exists during the whole shell growth. This cation exchange process may influence the homogeneous distribution of elements in the ZnS shell, and may generate new lattice mismatch in this case.

## Discussion

**Figure 8:**
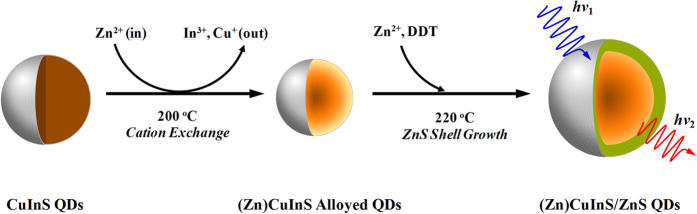
Proposed schematic mechanism of (Zn)CuInS/ZnS core/shell QDs formation. First, CuInS QDs with pyramidal chalcopyrite crystalline structure are synthesized at 215 °C (when Cu:In ≤ 1:2). Then, Cu^+^ and In^3+^ ions are partically replaced by Zn^2+^ in the CuInS QDs at 200 °C, leading to Cu and In defective (Zn)CuInS alloyed QDs. Last, coating ZnS shell onto (Zn)CuInS alloyed QDs to obtain (Zn)CuInS/ZnS core/shell QDs at 220 °C.

Currently, some experiments and models are used to explain the blinking mechanism of QDs^9^,^10^,^11^,^12^,^13^,^46^,^47^,^48^, and it is generally considered that the blinking behavior of QDs is mainly attributed to the nonradiative recombination process associated with traps on the surface of the QDs^9^,^21^,^49^. The proper surface passivation of QDs is an efficient way for suppressing QDs blinking by reducing the surface traps. Normally, a larger bandgap shell (such as CdS or ZnS shell) is introduced into the cores QDs surface (such as CdSe QDs)^13^,^22^,^25^. This method is mainly used to passivate the surface traps, but cannot be used to modify the interior traps of QDs. It is impossible to totally eliminate the blinking behavior of QDs. Recently, Klimov’s group found the fluorescence of CuInS/ZnS QDs originated from two channels^29^. The first channel stems from an internal defect state, such as a substitutional defect in which the I and III ions are swapped, and the second channel relates with surface traps of the nanocrystals. Therefore, eliminating the interior and exterior (surface) traps are both crucial to complete suppression of the blinking behavior. From the above experimental results and structural characterization, our strategy for suppressing the blinking behavior of QDs is schematically illustrated in Fig. 8. Firstly, the *in situ* interfacial alloying process is to eliminate the interior traps of QDs by forming an alloyed (Zn)CuInS QDs, and this step is very crucial for subsequently obtaining non-blinking and high photostability (Zn)CuInS/ZnS core/shell QDs^40^,^50^,^51^,^52^. Apart from forming a smooth band gap gradient, alloying also relaxes the lattice strain of unmatched crystal structures. The possibility for the cationic exchange process was due to these followings reasons: (1) the chalcopyrite structure of CuInS QDs is a lightly modified form of zinc blende, in which Zn^2+^ ion occupy the position of Cu^1+^ and In^3+^ ions; (2) Cu^1+^, Zn^2+^ and In^3+^ ions have a similar ionic radii of 60–62 pm in a four coordinate structure; (3) the crystal structures of CuInS and ZnS have similar lattice parameters of 0.540 and 0.552 nm, respectively; (4) CuInS and ZnS have a similar Gibbs formation enthalpy of −221 and −206 kJ/mol, respectively^53^,^54^. Therefore, Cu^1+^ and In^3+^ cations can be exchanged with Zn^2+^ ion due to thermodynamic driving force. Moreover, exchange reactions are allowed in nanometer-sized crystals at low temperature due to the effective reaction barrier of a diffusion controlled mechanism^41^,^55^.

In our experiments, the decrease in size was observed by HR-TEM analysis after the reaction of CuInS QDs with Zn^2+^ cation, which was one of the most striking indications of alloyed structure formation^27^,^29^,^38^,^41^,^44^. Previous studies illustrated that the reaction temperature played a main role in the decomposition kinetics of DDT, and further significantly affected the nucleation dynamics of CuInS QDs^12^,^21^,^26^,^27^,^28^,^29^. In our case, the annealing temperature (200 °C) for cation exchange reaction was lower than the nucleation temperature of CuInS QDs (215 °C) and the ZnS shell growth temperature (220 °C). Under this circumstance, the decomposition rate of DDT was significantly reduced and the cation exchange played a dominant role. In the ion exchange process, the original cations (Cu^1+^ and In^3+^) first diffuse out of the lattice, and then the free cations (Zn^2+^) rapidly occupy the formed vacancies to avoid the collapse of the lattice.

The quaternary alloyed structure of (Zn)CuInS QDs was further supported by comparing the XRD pattern of the CuInS QDs with those of the (Zn)CuInS QDs. By qualitative XRD analysis, we found that CuInS QDs (Cu:In stoichiometric ratios of 1:2 and 1:4) exhibited chalcopyrite structure (JCPDS file 32-0339). After treated with Zn^2+^ ion, the crystal structure of the (Zn)CuInS QDs (Cu:In:Zn stoichiometric ratios of 1:2:3 and 1:4:3) transformed into quaternary roquesite CuInZnS system (JCPDS file 47-1371, Fig. 5b). Because the sizes of the present (Zn)CuInS QDs were too small we cannot obtain the Zn distribution inside the nanocrystals by determining a compositional profile in the TEM. We speculated that the cation exchange first proceeded at the exterior of CuInS QDs, and then gradually permeated into the interior of the nanocrystals. That means the distribution of Cu^1+^ and In^3+^ gradually decreased from the core to the surface. In other words, these (Zn)CuInS alloyed QDs may possess a graded structure. The alloyed crystal structure reduced the rate of Auger recombination by softening the abrupt confinement potential of the (Zn)CuInS QDs^10^,^40^,^56^,^57^, and meanwhile greatly influenced the crystallinity and surface chemistry of these QDs^38^,^58^,^59^. The alloyed (quaternary) treatment of the QDs is an important process in repairing the defect-chemical state of the QDs. Some similar phenomenon was observed in the cation-exchanged Hg_x_Cd_1−*x*_Te QDs heterostructures, and those QDs were nearly free from lattice strain^60^. We believe that this *in situ* ion exchange process may play a key role in determining the crystal quality of the resulting (Zn)CuInS alloyed QDs, and is very crucial to preparing non-blinking QDs. Recently, Krauss’s group reported that the formation of the graded interface between materials comprising CdZnSe/ZnSe QDs could completely eliminate the QDs blinking^10^.

Secondly, the epitaxial deposition of ZnS shell on preformed (Zn)CuInS alloyed QDs is a significant interfacial engineering process for avoiding the generation of surface traps during the shell growth of QDs, and this procedure also improved the photostability of these non-blinking (Zn)CuInS/ZnS QDs. Here, we increase the annealing temperature up to 220 °C for ZnS shell growth, which is higher than the etching temperature (200 °C) of the alloyed (cation exchange) process of (Zn)CuInS QDs. Under higher temperature, the decomposition process of DDT plays the dominant role, this process can release the activated S by homolytic cleavage of the thiol and alkyl groups, and then the activated S and free Zn^2+^ consequently incorporates into the QDs^21^. It should be noted that the injection rate of the shell precursors were strictly controlled by a syringe pump^12^,^40^. This slow continuous shell precursor infusion and the relatively low reactivity of DDT provide an optimal condition, leading to a well-maintained particle size distribution during shell growth. This extremely slow growth rate of the ZnS shell layer is very critical for epitaxial growth at the alloyed core/multishell interface without lattice relaxation and crystalline defects^12^,^40^, considering that the lattice mismatch between (Zn)CuInS and ZnS is very small (less than 2%), because the lattice mismatch between CuInS and ZnS is about 2%^26^,^44^. Upon overcoating with ZnS as a higher band gap shell with an appropriate thickness, the surface of (Zn)CuInS alloyed QDs would be well passivated by the effective elimination of the surface defective sites, resulting in continuous emission of fluorescence.

The single-particle emission behavior of (Zn)CuInS/ZnS QDs (Cu:In:Zn stoichiometric ratios of 1:2:3) exhibit non-blinking behavior during the growth process of 20h. Measurements of elemental composition (ICP-OES and EDS) revealed that the main cation exchange (Zn^2+^ with Cu^1+^ and In^3+^) in the formation of (Zn)CuInS alloyed QDs was basically finished or reached equilibrium before shell growth (Supplementary Table S3). These data also indicated the molar ratios of Cu:In reached a relatively balanced level. Together with the slow injection of shell precursors, these (Zn)CuInS/ZnS QDs maintained an extremely low and relatively uniform growth rate during the whole growth process, which was proved by the size variations and size distributions of the TEM images (Fig. 6). Hence, the surface transformation and reconstruction of QDs were achieved with a slowly epitaxial growth of ZnS shell. This operation greatly reduced the new lattice mismatch, avoided the generation of surface traps, and the passivated QDs exhibited totally free of blinking behavior. Interestingly, the (Zn)CuInS/ZnS QDs (Cu:In:Zn stoichiometric ratio of 1:4:3) exhibit a transition from non-blinking to blinking during the shell growth process. Elemental analysis revealed that the cation exchange is an ongoing process during the whole ZnS shell growth, and this continuous cationic exchange process would lead to the inhomogeneous distribution of elements in the ZnS shell (Supplementary Table S4). Meanwhile, these QDs had faster growth rate, and their sizes were bigger than QDs with the stoichiometric ratio of 1:2:3 (Fig. 7). The lattice strain, which was caused by the lattice mismatch during the shell growth, probably led to the formation of dislocations and low-angle grain boundaries, and caused the growth to proceed incoherently. These defects may be the source of the formation of new nonradiative recombination sites within the ZnS shell^45^.

In summary, we described a new strategy for preparation of non-blinking (Zn)CuInS/ZnS QDs by precisely regulating the *in situ* interfacial alloying process of QDs. This strategy includes synthesis of CuInS QDs, forming (Zn)CuInS alloyed QDs, and coating ZnS shells onto (Zn)CuInS QDs to modify surface traps using thiol compounds as sulfur source and surface ligands. The suppressed blinking mechanism was mainly attributed to modifying QDs traps from interior to exterior by a step-by-step polishing strategy. We find that the stoichiometric ratios of the cationic precursors are crucial to control its single-particle PL emission behavior. The as-prepared non-blinking (Zn)CuInS/ZnS QDs consist of non-toxic elements, possess good optical properties and small particle size, and will become a promising optical materials for applications in displays and light-emitting devices or as fluorescent biological labels.

## Methods

### CuInS QDs synthesis

For a representative synthesis of CuInS QDs (standard reaction), 0.019 g CuI powder, 0.1168 g In(Ac)_3_, and 10 mL 1-dodecanethiol (DDT) were mixed in a 100 mL three-neck flask. The round-bottom flask was first put under vacuum and heated to 80 °C while stirring, backfilled with Ar. Under argon flux, the reaction temperature was increased to about 215 °C with a rate of 20 °C/min for 1–60 min, dependent on the desired size. The heating element was then removed and the QDs are allowed to cool. Upon heating up, the reaction mixture became transparent and colorless at around 160 °C, and the solution color started to change at 190 °C, from yellow to red and then dark red at 215 °C, which indicated the nucleation and growth of CuInS QDs. In order to synthesize CuInS QDs with different stoichiometric ratios (that is, with a ratio of copper to indium lower than one), the amount of CuI (0.1 mmol) and DDT (10 mL) were held fixed, while the dosage of In(Ac)_3_ was adjusted. Without purification, the crude solution was used for the synthesis of the (Zn)CuInS alloyed QDs.

### Preparation of (Zn)CuInS alloyed QDs

(Zn)CuInS alloyed QDs were prepared by *in situ* zinc cation exchange of CuInS QDs. The zinc precursor solution was prepared by dissolving 0.1897 g of zinc stearate in 4 mL of ODE and 1 mL TOP. The CuInS QDs solution was degassed at 120 °C for 30 min. Without intermediate purification step of the CuInS QDs, the zinc precursor solution was added dropwise by a syringe pump within 30 min to the CuInS QDs at 120 °C. Then, the temperature was heated to about 200 °C and maintained for 90 min before cooling to 120 °C.

### Epitaxial shell growth

The (Zn)CuInS alloyed QDs mixture was degassed at 120 °C under vacuum for 30 min to remove volatiles added or produced during the reaction. Under argon flux, the temperature was increased to about 220 °C, and a solution of zinc and sulfur mixed precursors (20 mL of a 0.1 M solution of zinc stearate in 5 mL oleic acid, 5 mL DDT and 10 mL ODE) were injected by a syringe pump at a rate of 2 mL/hr. The aliquots were taken out at a certain time for further measurements. The entire growth process was monitored by UV-vis and photoluminescence spectroscopy. After the injection of zinc and sulfur mixed precursor, the solution was stirred for an additional 60 min of refluxing process at 220 °C, then the heating vessel was allowed to naturally cool to the room temperature.

### Preparation of glass substrates

In our method, a glass substrate was treated with an oxidizing solution of sulfuric acid and hydrogen peroxide (“piranha etch”), so as to remove any organic contaminants and promote the formation of hydroxyl groups on the glass surface, making the substrate hydrophilic. Glass coverslips with a thickness of 0.1 mm were purchased from Fisher (24 × 40 × 1 mm^3^, U.S.A). These glass substrates were washed with Millipore water before use and dried for 2 h in an oven. Piranha solution was prepared by mixing 98% H_2_SO_4_ with H_2_O_2_ (3:1, v/v). Each washed glass coverslip was immersed into the freshly made solution of piranha etch and left for 30 min. Then, the glass slides were removed, dipped in pure Millipore water and sonicated in a sonic bath for 15 min. This procedure was conducted 5 times. The coverslips were dried using a hair dryer.

### Morphological and structural characterization of QDs

For structural and size characterization, X-ray powder diffraction (XRD) spectra were taken on a D/max-Rbusing 2550VL/PC X-ray diffractometer (Rigaku, Japan) equipped with Cu K_*α*_ radiation (*λ* = 1.5418 Å, 40 kV, 30 mA). HRTEM images were recorded on a JEM-2100 (JEOL Ltd., Japan) with an accelerating voltage of 200 kV. The average QD size and size distribution were estimated by analyzing HRTEM images of a large number of QDs. The actual compositions of CuInS QDs were analyzed by an iCAP-6300 inductively coupled plasma optical emission spectrometer (Thermo Ltd., U.S.A.). All chemical analyses performed by ICP-OES were affected by a systematic error of about 5%. Samples were dissolved in HCl/HNO_3_ 3:1 (v/v). EDS were performed using an energy-dispersive X-ray spectroscopy equipped on a JEOL-2100F (JEOL Ltd., Japan) transmission electron microscope.

### Optical measurement of QDs at ensemble level

Absorption spectra of CuInS QDs, (Zn)CuInS alloyed QDs and (Zn)CuInS/ZnS QDs were obtained by a UV/Vis-3501 spectrophotometer and fluorescence emission spectra were recorded with an F-380 spectrometer (Tianjin Gangdong SCI & Tech. Development Co., Ltd., China). The relative PL quantum yields (QYs) of various QDs samples were comparatively studied by comparison with that of Rhodamine 6G (95%) according to the method described in the reference (in the supplementary information). Absorption and PL emission spectra were measured twice, and average QY was recorded.

### Optical measurement of QDs at single-particle level

In individual nanoparticle experiment, the samples were prepared by spin casting freshly diluted newly-prepared QDs in mixed organic solvent (90% hexane-10% octane). The QDs density on the substrate was controlled by changing the concentration of the QDs in the solution before spin coating.

Fluorescence trajectories of single QDs were acquired by a total internal reflection fluorescence microscopy (TIRFM) imaging system based on an Olympus IX 71 inverted fluorescence microscope (Olympus Optical Co., Japan). The schematic diagram of the TIRFM setup was described in the supplementary information. The QDs samples were excited with 488 nm argon ion laser (ILT Ion Laser Technology, Shanghai, China) and the laser power monitored in front of the microscopy objective (NA1.45/60×, Olympus Optical Co., Japan) was measured to be 0.28 mW after it was attenuated. Fluorescence from the sample was collected by the same objective, separated from the excitation light by a dichroic mirror and emission filters, and then focused into an electron-multiplying charge coupled device (EM-CCD) camera with a frame-transfer device (Evolve 512, Photometrics, U.S.A). The frame transfer device has an imaging array of 512 × 512 pixel with 16 × 16 *μ*m^2^/pixel. As the image can be quickly transferred from the sensor area to the frame transfer area, there is no need for a mechanical shutter. When operated in overlap mode, the camera is extremely useful in applications requiring continuous imaging (100% duty cycle) and can capture all the light emitted from the samples. The image acquisition, single spot trace extraction and data were processed using ImageJ software (National Institutes of Health, free software, http://rsbweb.nih.gov/ij/index.html), and MATLAB (The MathWorks Inc., U.S.A.). All measurements were performed at room temperature. Note that we used the average gray value in the analysis area around the QD as the fluorescence signal of the QD to avoid possible problem related to the light diffusion. The background, estimated as the average intensity in the vicinity of every dot at a given excitation power, was subtracted from the fluorescence signal. The exposure time was set at 50 ms for each frame in all the samples. This method was used to image and record the PL intensity variation of many single dots once.

## Additional Information

**How to cite this article**: Zhang, A. *et al.* Non-blinking (Zn)CuInS/ZnS Quantum Dots Prepared by *In Situ* Interfacial Alloying Approach. *Sci. Rep.* **5**, 15227; doi: 10.1038/srep15227 (2015).

## Supplementary Material

Supplementary Information

Supplementary Video 1

Supplementary Video 2

Supplementary Video 3

Supplementary Video 4

Supplementary Video 5

Supplementary Video 6

Supplementary Video 7
