# Supplementary material for: Non-blinking (Zn)CuInS/ZnS Quantum Dots Prepared by In Situ Interfacial Alloying Approach
Source: Sci Rep. 2015 Oct 13;5:15227. doi: 10.1038/srep15227 (PMC4602315; doi:10.1038/srep15227)
Supplement: Supplementary Information [file srep15227-s1.pdf]

Supplementary Information

Non-blinking (Zn)CuInS/ZnS Quantum Dots Prepared by In Situ Interfacial Alloying Approach

Aidi Zhang[1,†], Chaoqing Dong[1,†*], Liang Li[2,†], Jinjin Yin[1], Heng Liu[1], Xiangyi Huang[1] & Jicun Ren[1,*]

[1]School of Chemistry and Chemical Engineering, State Key Laboratory of Metal Matrix Composites, Shanghai Jiao Tong University, 800 Dongchuan Road, Shanghai 200240, China.

[2]School of Environmental Science and Engineering, Shanghai Jiao Tong University, 800 Dongchuan Road, Shanghai 200240, China.

[†]These authors contributed equally to this work.

Correspondence and requests for materials should be addressed to C.Q.D. (email: cqdong@sjtu.edu.cn) or J.C.R. (email: jicunren@sjtu.edu.cn)

Supplementary Experimental Section

The measurement of quantum yields

The PL quantum yields (QY) of various samples were measured by using Rhodamine 6G (R6G) as a reference fluorescent dye with the known QY (95%) and comparing the integrated fluorescence intensity of the solutions, both record exciting samples having the same absorbance (<0.1 au in order to minimize possible reabsorption effects). The PL QYs of the as-prepared QDs were calculated using the following equations:

$$QY_{sample}=QY_{R6G}\times(grad_{sample}/grad_{R6G})\times(n_{sample}/n_{R6G})^2$$

Where grad stands for the gradient (slope) of the plot of the integrated fluorescence intensity *vs* absorbance, and n stands for the refractive index of the solvent (1.36 for ethanol, 1.37 for hexane, and 1.50 for toluene). The excitation wavelength was set at 450 nm. PL QYs of the QDs were calculated by comparing their integrated emissions with that of Rhodamine 6G (QY of ～95%) ethanol solution with an identical optical density of ～0.05 at 450 nm[1-3].

The determination of single-particle emissions

In this study, we design two feasibility scenarios to identify the detected light spot came from a single dot. Firstly, the dispersion procedure of single particles is feasible. In the individual nanoparticle detection experiment, the good dispersibility of the QDs is the necessary prerequisite for the total internal reflection fluorescence imaging. We try to prepared nanocrystal samples by spin-casting a dilute solution of CuInS based QDs diluted in a chloroform/poly(methylmethacrylate) solution (PMMA, the concentration range from 0.5%-6.0% gmL^{-1}). Unfortunately, the QDs were seriously aggregated and we cannot get uniform light spot imaging (Supplementary Fig. 30). Pure organic solvent (toluene) or mixed organic solvent (90% hexane-10% octane) was used in single particle disperse system for blinking behavior studies[4]. In an open environment without isolating material, the luminescent process of a single QD was detected under a TIRFM. This process can rule out the possibility of PMMA spheres during the spray of polymer solution over the pretreated glass substrate[5]. Thankfully, individual QDs were shown to be homogeneously dispersed in the fluorescence images, and no aggregation was observed in this cases. We observed that the (Zn)CuInS alloyed QDs synthesized with different stoichiometric ratios of cations exhibited blinking or non-blinking behavior. It should be noted that the blinking behavior is an important feature of single individual nanoparticle[6]. This result also indicated that our dispersion procedure has good generality for single particle detection of thiol-modified QDs.

At the same time, we explored thiol-modified CdSe/CdS QDs with similar size

and surface ligands composition to assess the reliability of our dispersion solutions. Thiol-modified CdSe/CdS QDs were prepared by using our previous method[3]. These QDs at least were covered with 1-dodecanethiol as surface ligand layer. The QDs were well separated and no aggregation is observed in this case (Supplementary Fig. 31a). These QDs exhibit severe blinking and long periods of off time (Supplementary Fig. 31b). This result further indicates that our dispersion procedure has good generality for single particle detection of thiol-modified QDs.

Secondly, to investigate in more detail the PL emission behavior of single QDs under different lasers, we studied their fluorescence emission with a 405 nm continuous-wave laser and a 450 nm pulse laser (see the following description for more details), using a home-built epi-illumination fluorescence microscope system equipped with a Zeiss 63 × oil immersion objective (numerical aperture = 1.46) and suitable spectral filters[7]. Detected sample were used (Zn)CuInS/ZnS QDs (Cu:In:Zn stoichiometric ratio of 1:2:3) with ZnS reaction time of 20 h in which all macroscopic variables are identical except the nature of laser excitation. Surprisingly, the QDs exhibit continuous fluorescent emission under 450 pulse laser (Supplementary Fig. 32a, Video S6). However, after we changed the laser source into 405 nm continuous-wave laser for the same detected area (the same imaging area of the same coverslip which coated with the same QDs), the QDs exhibit blinking to some extent (Supplementary Fig. 32b, Video S7). These two different phenomena reflect indirectly that the single light spot came from a single QD. This also means our new QDs also have sensitivity with laser sources at single-pot level.

The detailed information for the 405 nm continuous-wave laser and 450 pulse laser tests was described as follows[7]: The single-dot optical measurements were performed using a home-built epi-illumination fluorescence microscope system equipped with a Zeiss 63 × oil immersion objective (numerical aperture = 1.46) and suitable spectral filters. The excitation light source for the PL intensity time traces was a 405 nm continuous-wave laser and 450 nm pulse laser. The PL intensity time traces of single dots were recorded by an Andor DU-897 EMCCD. For PL intensity measurements, movies with exposure time of 100 ms per frame were recorded for designated time length. The emission intensity of each QD on each frame was determined by the mean of the gray value of 8 × 8 pixel (16 × 16 μm^2/pixel) square containing the whole emission spot of the dot in all frames. The corresponding background intensity was determined by the mean of the gray values in a square of the same size closed to the QD but without any emission signal from visible QDs. These measurements were preformed in Zhejiang University (Hangzhou, China).

Additionally, we tried to test the second-order photon correlation (or antibunching) experiments performed with a time-correlated single-photon counting system to confirm that all measurements were associated with a single dot[7]. Unfortunately, the signal to noise ratio of (Zn)CuInS/ZnS QDs was not high enough to obtain antibunching curves even though the QDs exhibited good photostability. This is because that the size of (Zn)CuInS/ZnS QDs is smaller and their BPP (brightness per particle) is lower when compared to CdSe/CdS QDs. These measurements were preformed in Zhejiang University (Hangzhou, China).

Supplementary Movies List

Here seven videos are included in the manuscript.

Supplementary Video 1. The video for (Zn)CuInS alloyed QDs (the stoichiometric ratio of Cu:In:Zn is 1:2:3) excited by a continuous 488 nm Argon ion laser. The temporal resolution is 50 ms.

Supplementary Video 2. The video for (Zn)CuInS alloyed QDs (the stoichiometric ratio of Cu:In:Zn is 1:1:3) excited by a continuous 488 nm Argon ion laser. The temporal resolution is 50 ms.

Supplementary Video 3. The video for (Zn)CuInS/ZnS QDs (the stoichiometric ratio of Cu:In:Zn is 1:2:3) with ZnS shell growth time of 10 h. These QDs were excited by a continuous 488 nm Argon ion laser. The temporal resolution is 50 ms.

Supplementary Video 4. The video for (Zn)CuInS/ZnS QDs (the stoichiometric ratio of Cu:In:Zn is 1:4:3) with ZnS shell growth time of 10 h. These QDs were excited by a continuous 488 nm Argon ion laser. The temporal resolution is 50 ms.

Supplementary Video 5. The video for (Zn)CuInS/ZnS QDs (the stoichiometric ratio of Cu:In:Zn is 1:4:3) with ZnS shell growth time of 20 h. These QDs were excited by a continuous 488 nm Argon ion laser. The temporal resolution is 50 ms.

Supplementary Video 6. The video for (Zn)CuInS/ZnS QDs (the stoichiometric ratio of Cu:In:Zn is 1:2:3) with ZnS shell growth time of 20 h. These QDs were excited by a 450 nm pulse laser. The temporal resolution is 100 ms.

Supplementary Video 7. The video for (Zn)CuInS/ZnS QDs (the stoichiometric ratio of Cu:In:Zn is 1:2:3) with ZnS shell growth time of 20 h. These QDs were excited by a 405 nm continuous-wave laser. The temporal resolution is 100 ms.

Supplementary Figures

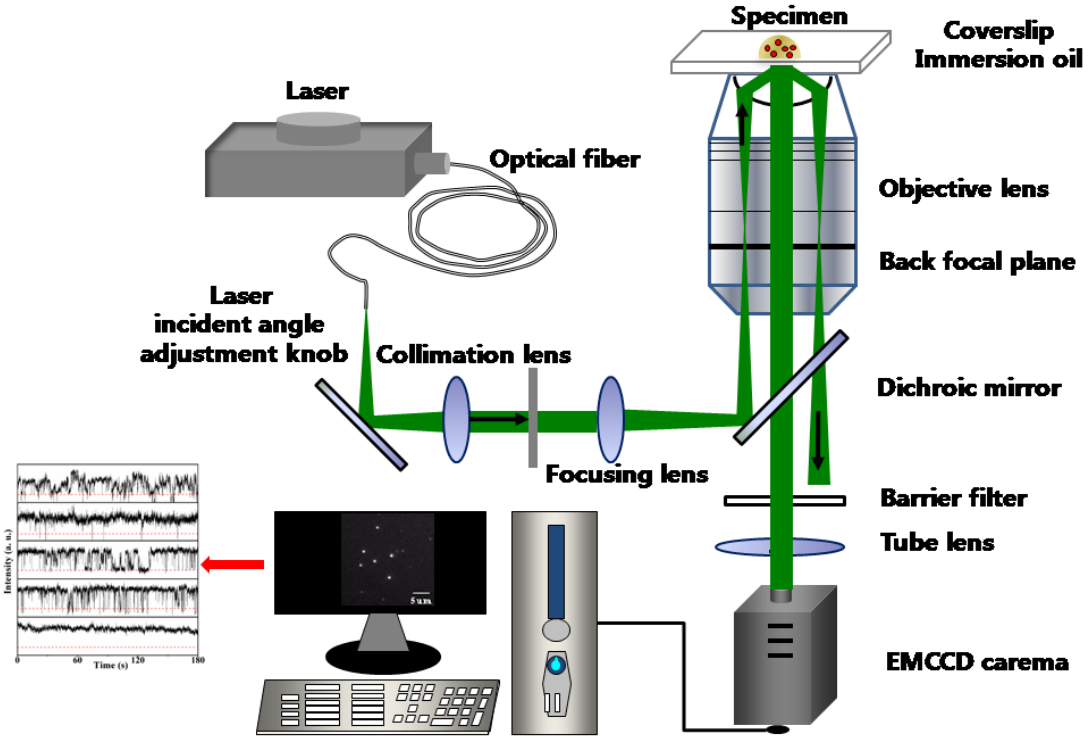

Supplementary Figure 1. Schematic diagram of Total Internal Reflection Fluorescence Microscopy (TIRFM) setup.

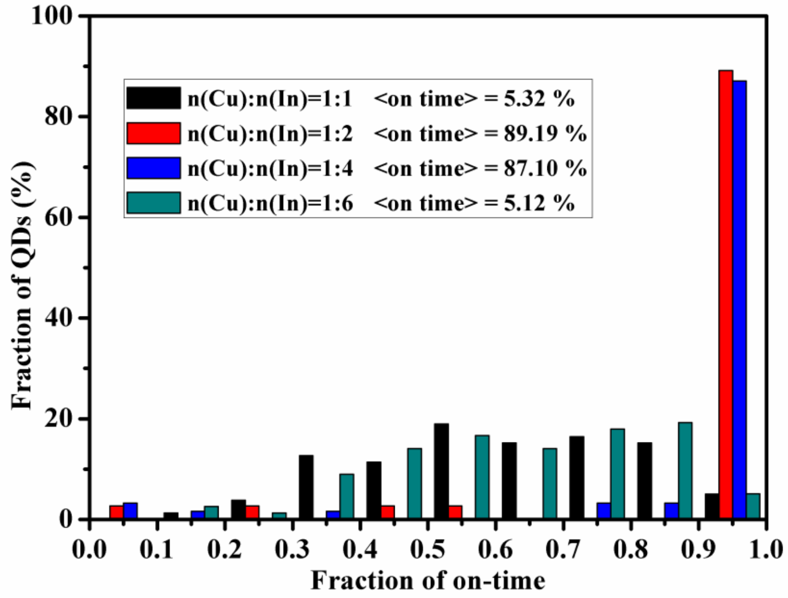

Supplementary Figure 2. Histograms of “on time” distribution fraction of (Zn)CuInS alloyed QDs (the stoichiometric ratios of Cu:In:Zn are 1:1:3, 1:2:3, 1:4:3, and 1:6:3, respectively) constructed from analysis of intensity trajectories of single dots. More than 100 dots are randomly selected and measured for each QDs sample.

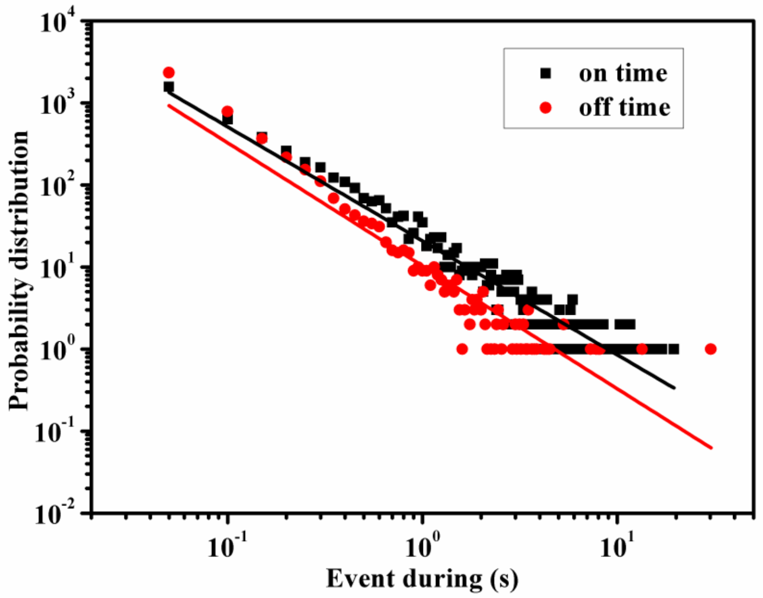

Supplementary Figure 3. Log-log plot of the probability densities of "on time" and "off time" for (Zn)CuInS alloyed QDs (the stoichiometric ratio of Cu:In:Zn is 1:1:3). The straight lines represent a power-law fitting using the equation of $P(t_{on/off}) = Bt^{-m_{on/off}}$, where the black line for "on time" and the red line for "off time".

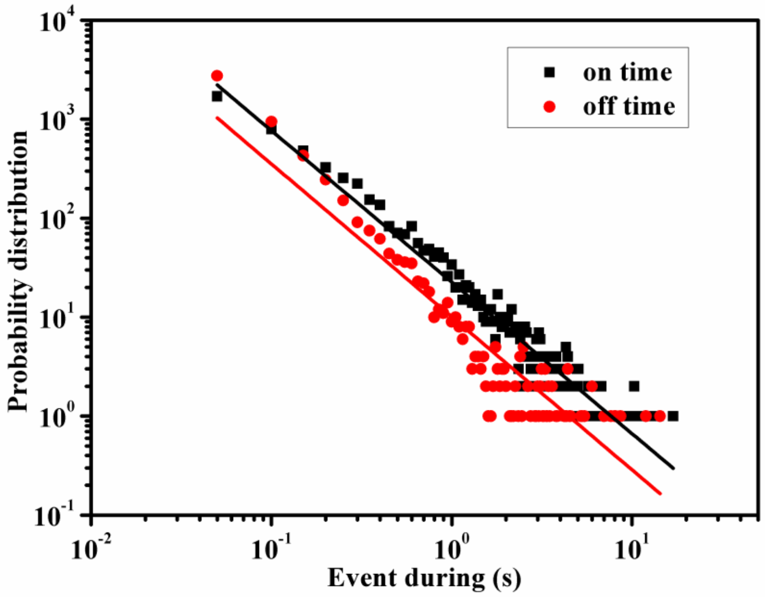

Supplementary Figure 4. Log-log plot of the probability densities of "on time" and "off time" for (Zn)CuInS alloyed QDs (the stoichiometric ratio of Cu:In:Zn is 1:6:3). The straight lines represent a power-law fitting using the equation of $P(t_{on/off}) = Bt^{-m_{on/off}}$, where the black line for "on time" and the red line for "off time".

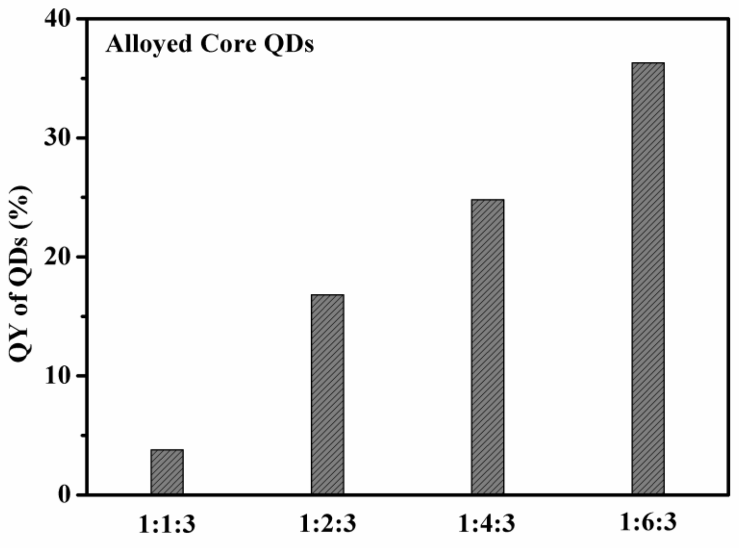

Supplementary Figure 5. PL QYs for (Zn)CuInS alloyed QDs with different stoichiometric ratios of Cu, In and Zn (1:1:3, 1:2:3, 1:4:3, and 1:6:3, respectively).

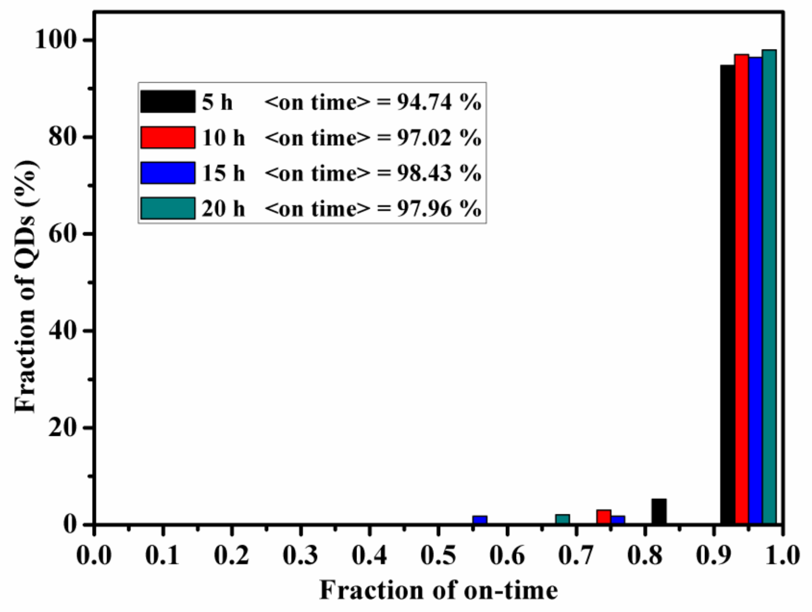

Supplementary Figure 6. Histograms of "on time" distribution fraction of (Zn)CuInS/ZnS core/shell QDs with different ZnS shell growth time (the stoichiometric ratio of Cu:In:Zn is 1:2:3) constructed from analysis of intensity trajectories of single dots. More than 100 dots are randomly selected and measured for each QDs sample.

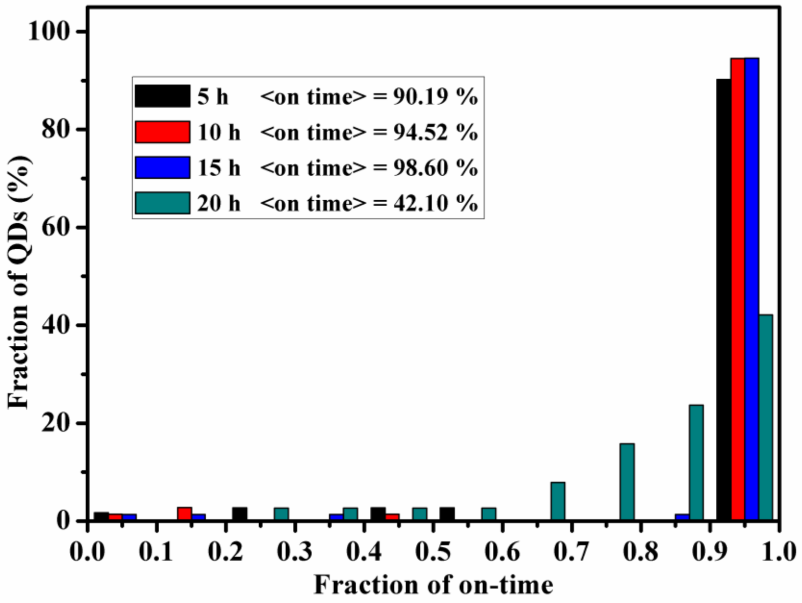

Supplementary Figure 7. Histograms of "on time" distribution fraction of (Zn)CuInS/ZnS core/shell QDs with different ZnS shell growth time (the stoichiometric ratio of Cu:In:Zn is 1:4:3) constructed from analysis of intensity trajectories of single dots. More than 100 dots are randomly selected and measured for each QDs sample.

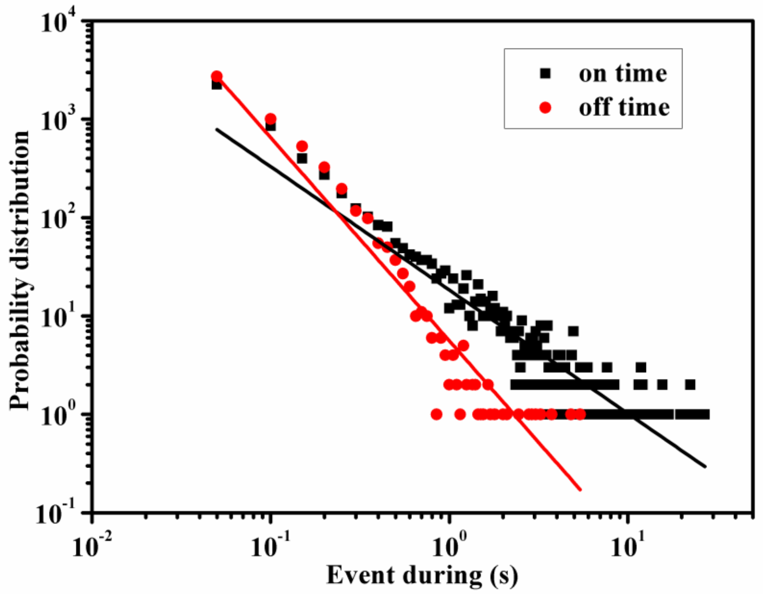

Supplementary Figure 8. Log-log plot of the probability densities of "on time" and "off time" for (Zn)CuInS/ZnS core/shell QDs (the stoichiometric ratio of Cu:In:Zn is 1:4:3) with ZnS growth time of 20 h. The straight lines represent a power-law fitting using the equation of $P(t_{on/off}) = Bt^{-m_{on/off}}$, where the black line for "on time" and the red line for "off time".

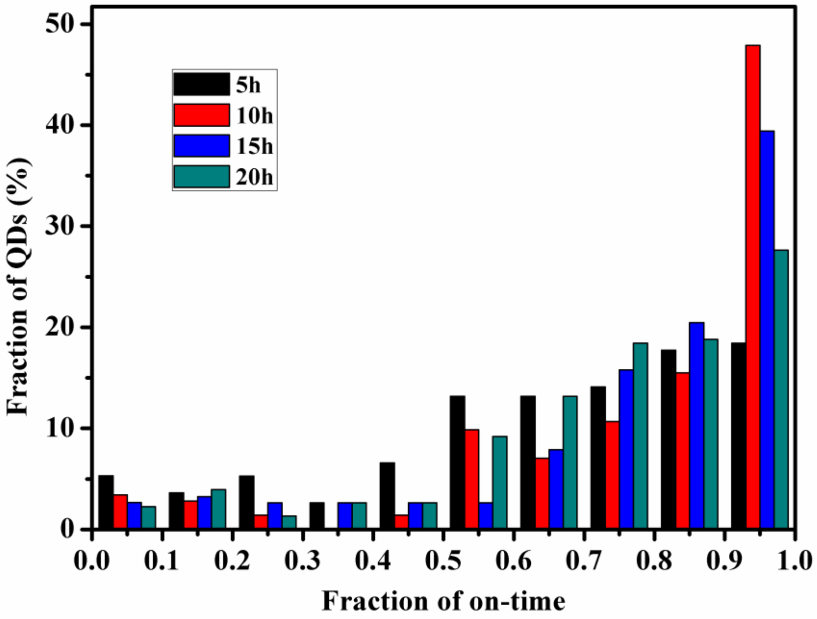

Supplementary Figure 9. Histograms of "on time" distribution fraction of (Zn)CuInS/ZnS core/shell QDs with different ZnS shell growth time (the stoichiometric ratio of Cu:In:Zn is 1:1:3) constructed from analysis of intensity trajectories of single dots. More than 100 dots are randomly selected and measured for each QDs sample.

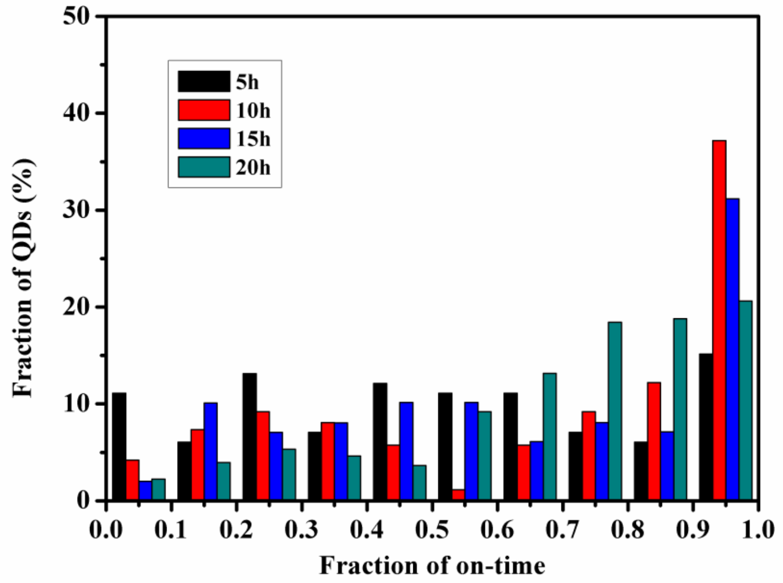

Supplementary Figure 10. Histograms of "on time" distribution fraction of (Zn)CuInS/ZnS core/shell QDs with different ZnS shell growth time (the stoichiometric ratio of Cu:In:Zn is 1:6:3) constructed from analysis of intensity trajectories of single dots. More than 100 dots are randomly selected and measured for each QDs sample.

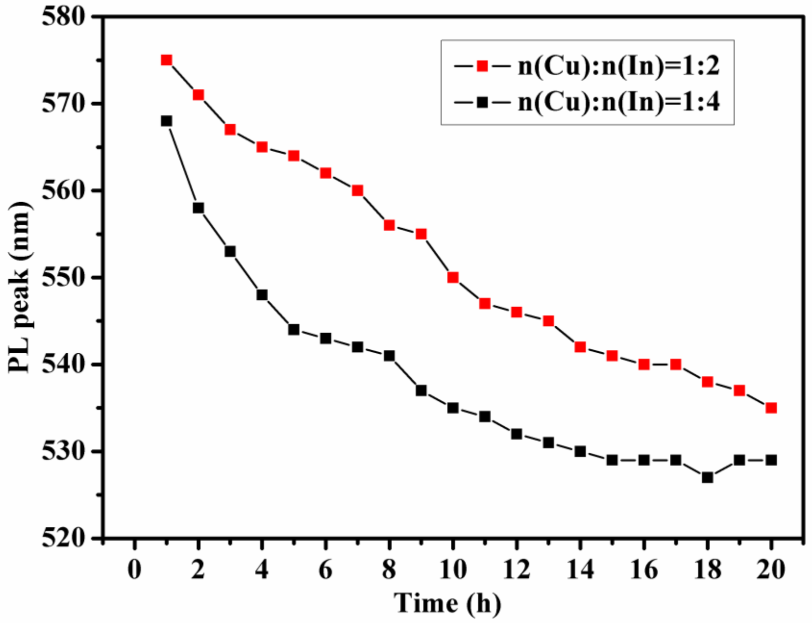

Supplementary Figure 11. Temporal evolution of PL peaks for (Zn)CuInS/ZnS core/shell QDs (the stoichiometric ratios of Cu:In:Zn are 1:2:3 and 1:4:3) prepared with different ZnS shell growth time.

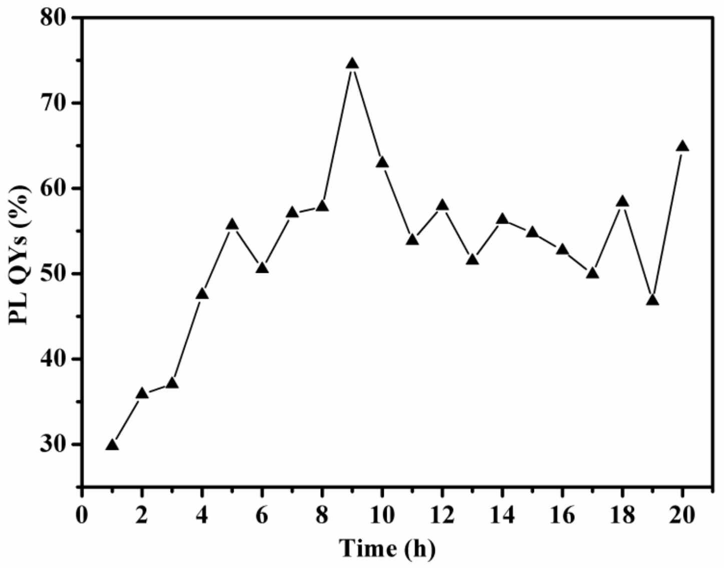

Supplementary Figure 12. PL QYs for (Zn)CuInS/ZnS core/shell QDs (the stoichiometric ratio of Cu:In:Zn is 1:2:3) prepared with different ZnS shell growth time.

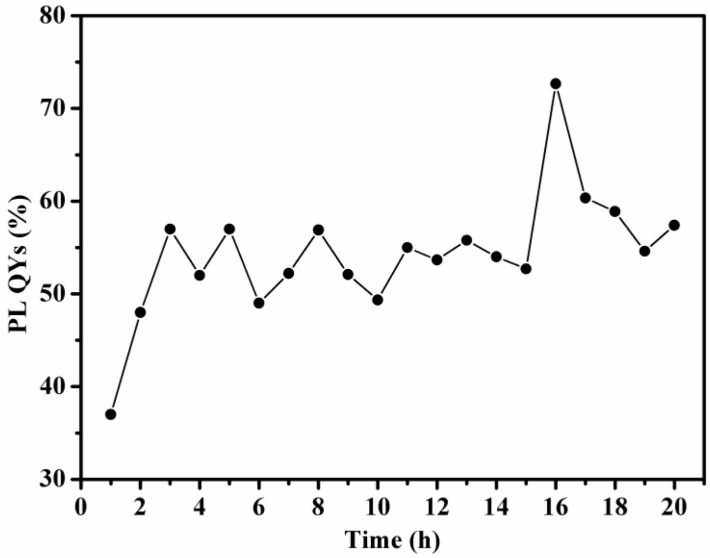

Supplementary Figure 13. PL QYs for (Zn)CuInS/ZnS core/shell QDs (the stoichiometric ratio of Cu:In:Zn is 1:4:3) prepared with different ZnS shell growth time.

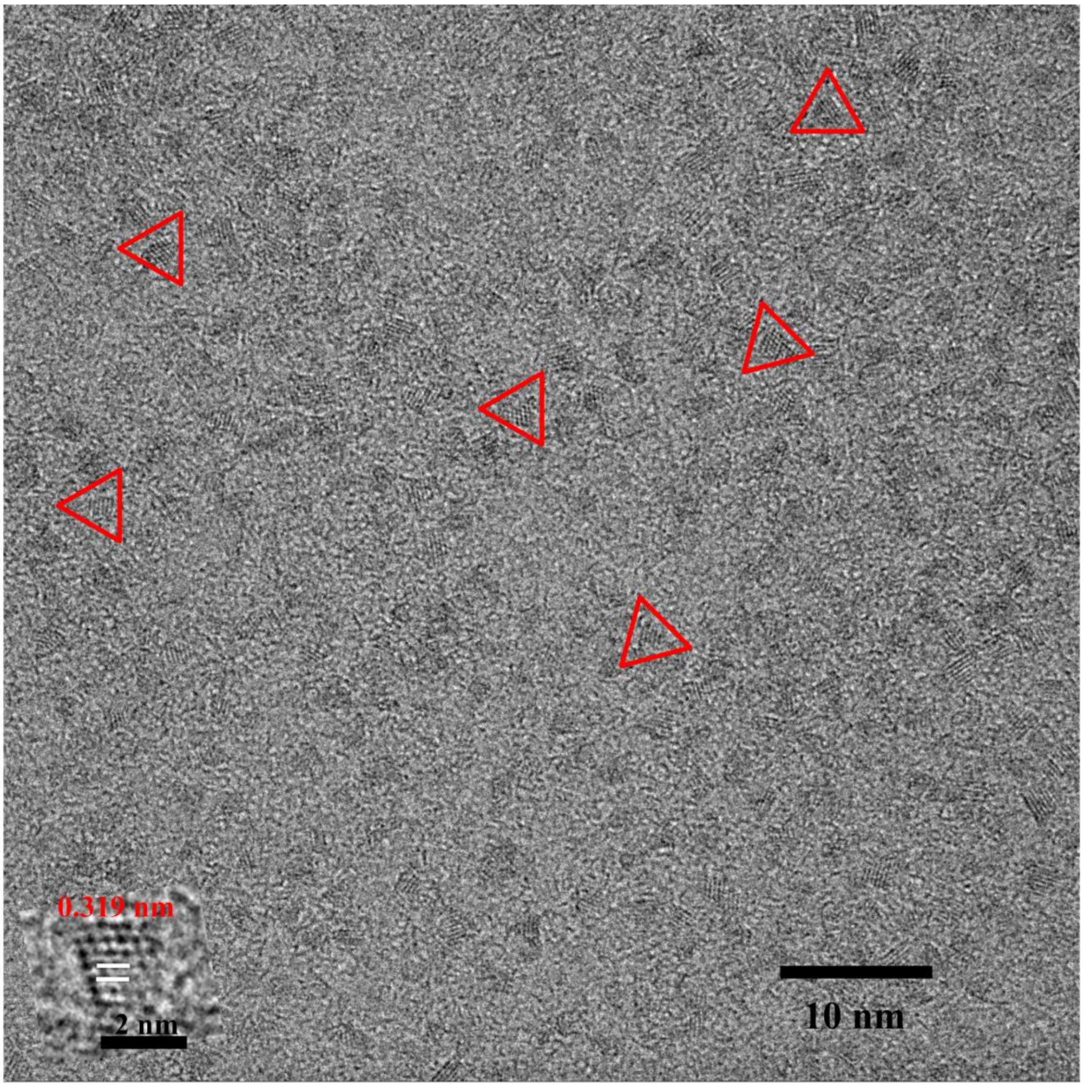

Supplementary Figure 14. A representative HR-TEM image of CuInS QDs (the stoichiometric ratio of Cu:In is 1:2) with reaction time of 30 min. The red triangle frames indicate the shapes of the CuInS QDs.

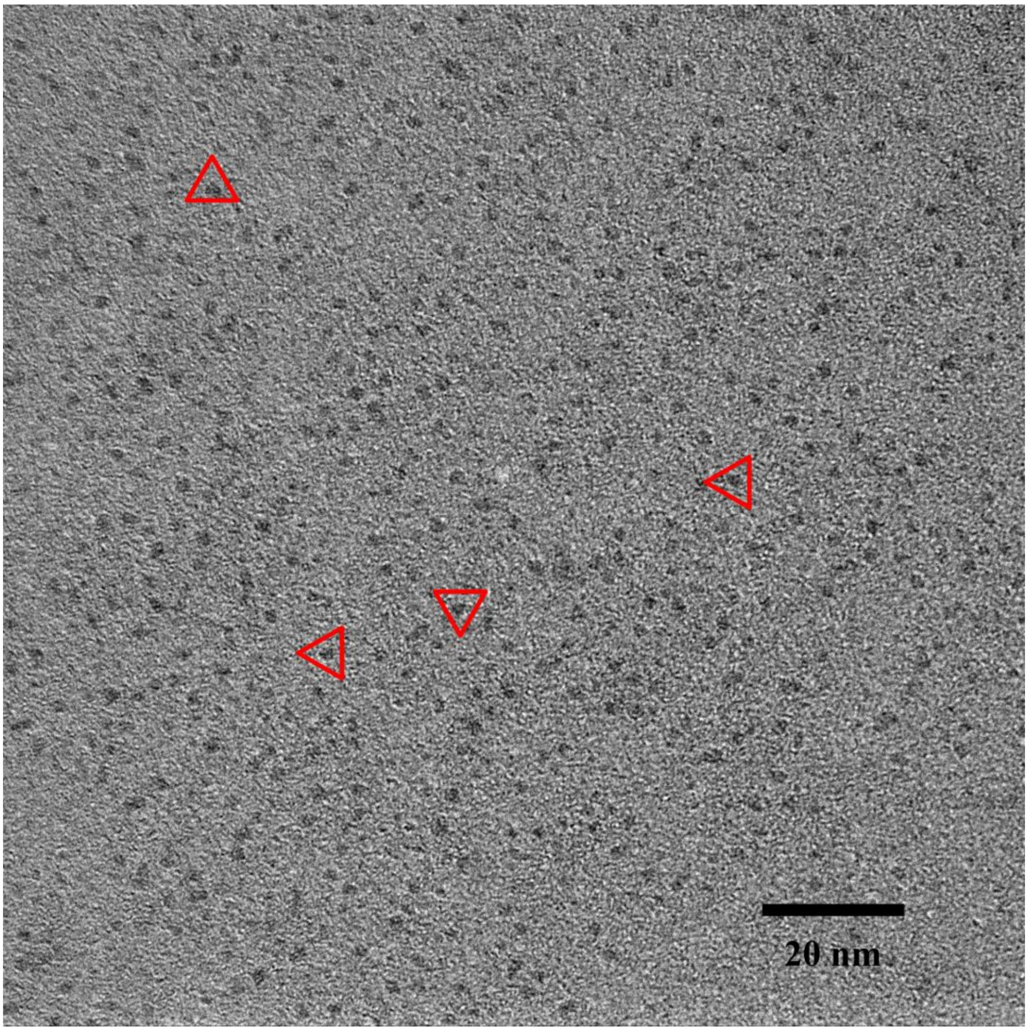

Supplementary Figure 15. A representative TEM image of CuInS QDs (the stoichiometric ratio of Cu:In is 1:4) with reaction time of 30 min. The red triangle frames indicate the shapes of the CuInS QDs.

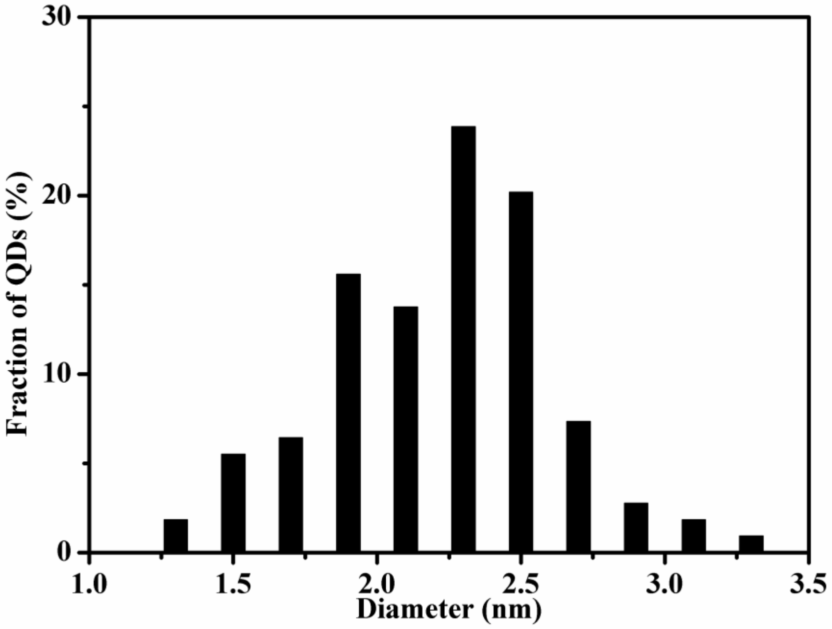

Supplementary Figure 16. Particle distribution histograms for CuInS QDs (the stoichiometric ratio of Cu:In is 1:2) with reaction time of 30 min. To build the histograms, the longest dimension for over 200 particles in each sample has been measured. The average diameter of QDs is about 2.1 nm.

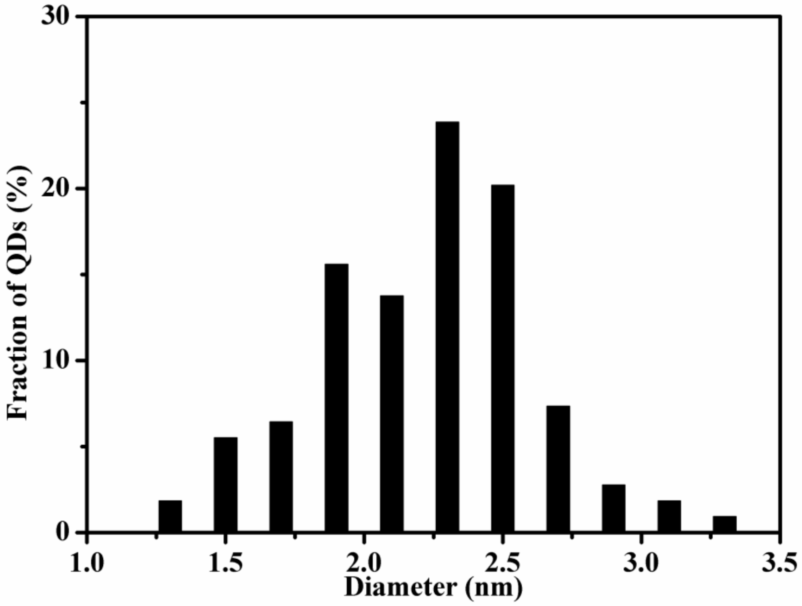

Supplementary Figure 17. Particle distribution histograms for CuInS QDs (the stoichiometric ratio of Cu:In is 1:4) with reaction time of 30 min. To build the histograms, the longest dimension for over 200 particles in each sample has been measured. The average diameter of QDs is about 2.2 nm.

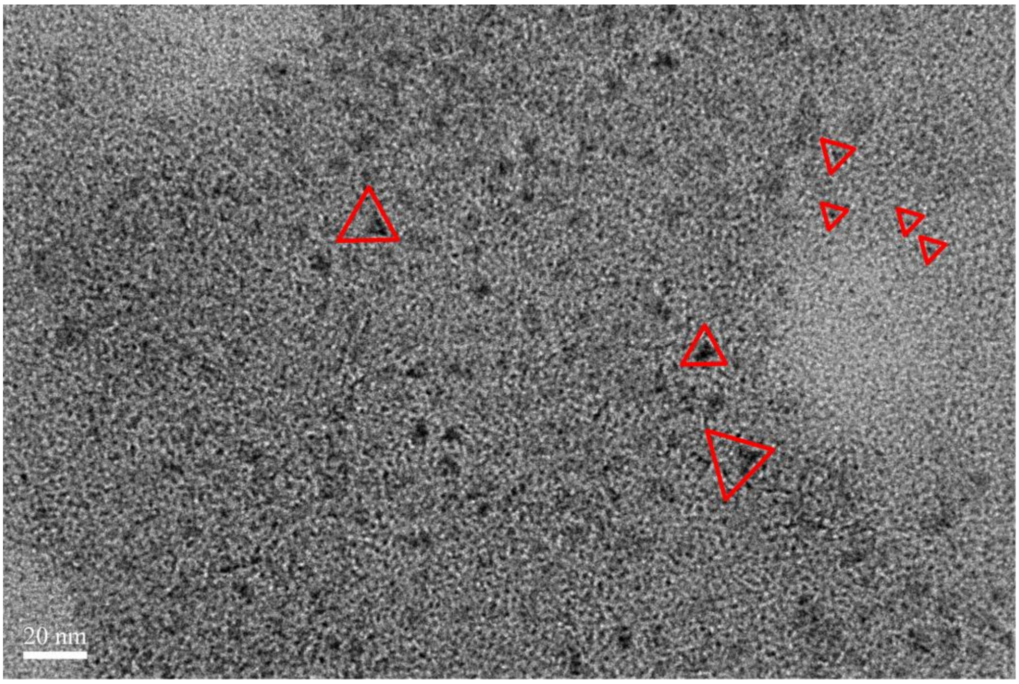

Supplementary Figure 18. A representative TEM image of CuInS QDs (the stoichiometric ratio of Cu:In is 1:1) with reaction time of 30 min. The red triangle frames indicate the shapes of the CuInS QDs. These QDs have wide size distribution of about 2.0-5.0 nm.

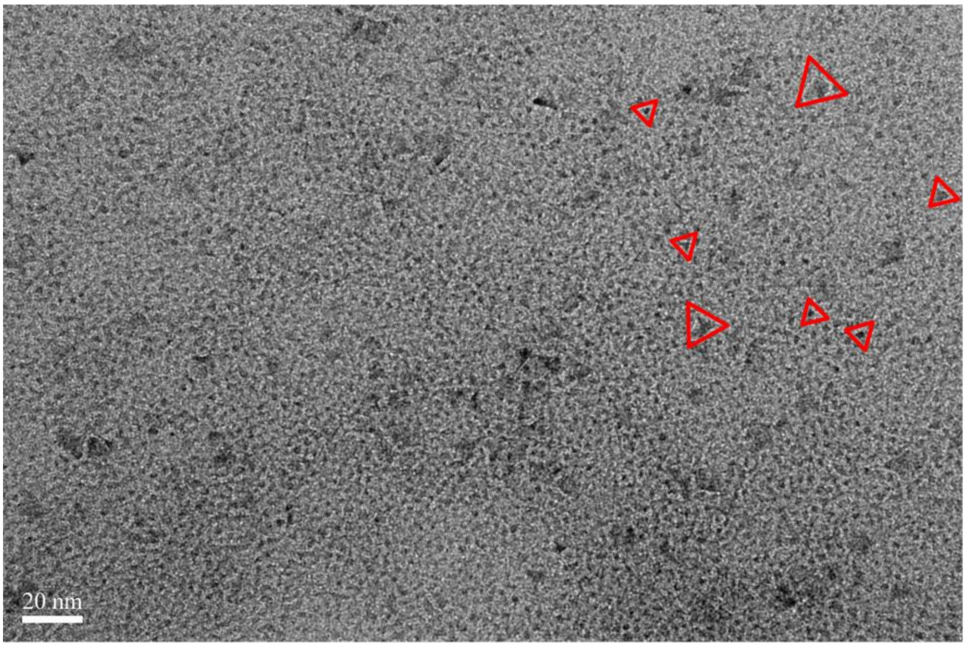

Supplementary Figure 19. A representative TEM image of CuInS QDs (the stoichiometric ratio of Cu:In is 1:6) with reaction time of 30 min. The red triangle frames indicate the shapes of the CuInS QDs. These QDs have wide size distribution of about 2.0-5.0 nm.

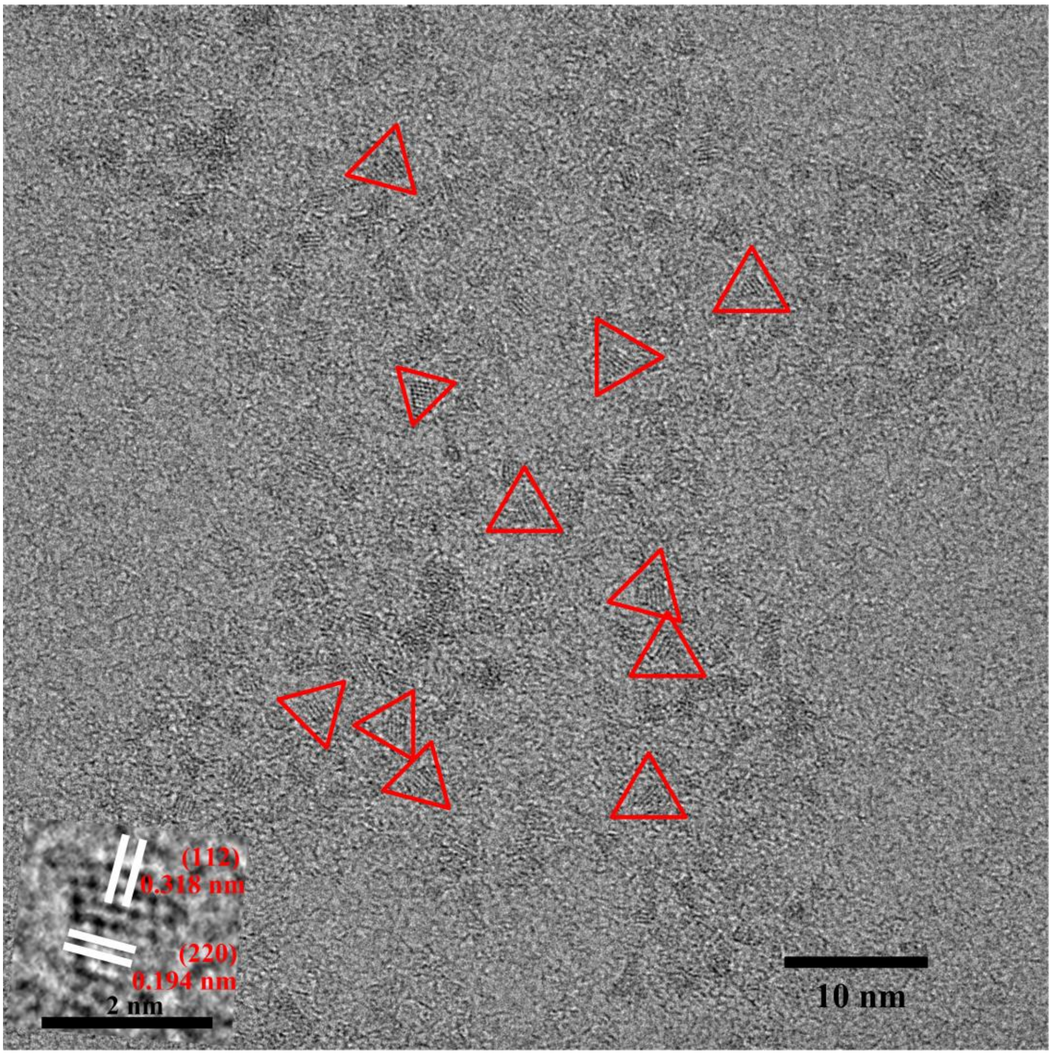

Supplementary Figure 20. A representative TEM image of (Zn)CuInS alloyed QDs (the stoichiometric ratio of Cu:In:Zn is 1:2:3) with etching time of 90 min. The red triangle frames indicate the shapes of the (Zn)CuInS alloyed QDs. The insert is a HR-TEM image of a representative (Zn)CuInS alloyed QDs and this QD exhibits both the typical (112) and (220) lattice sets compatible with the chalcopyrite-like structure, with *d*-spacing of 0.318 nm and 0.194 nm, respectively.

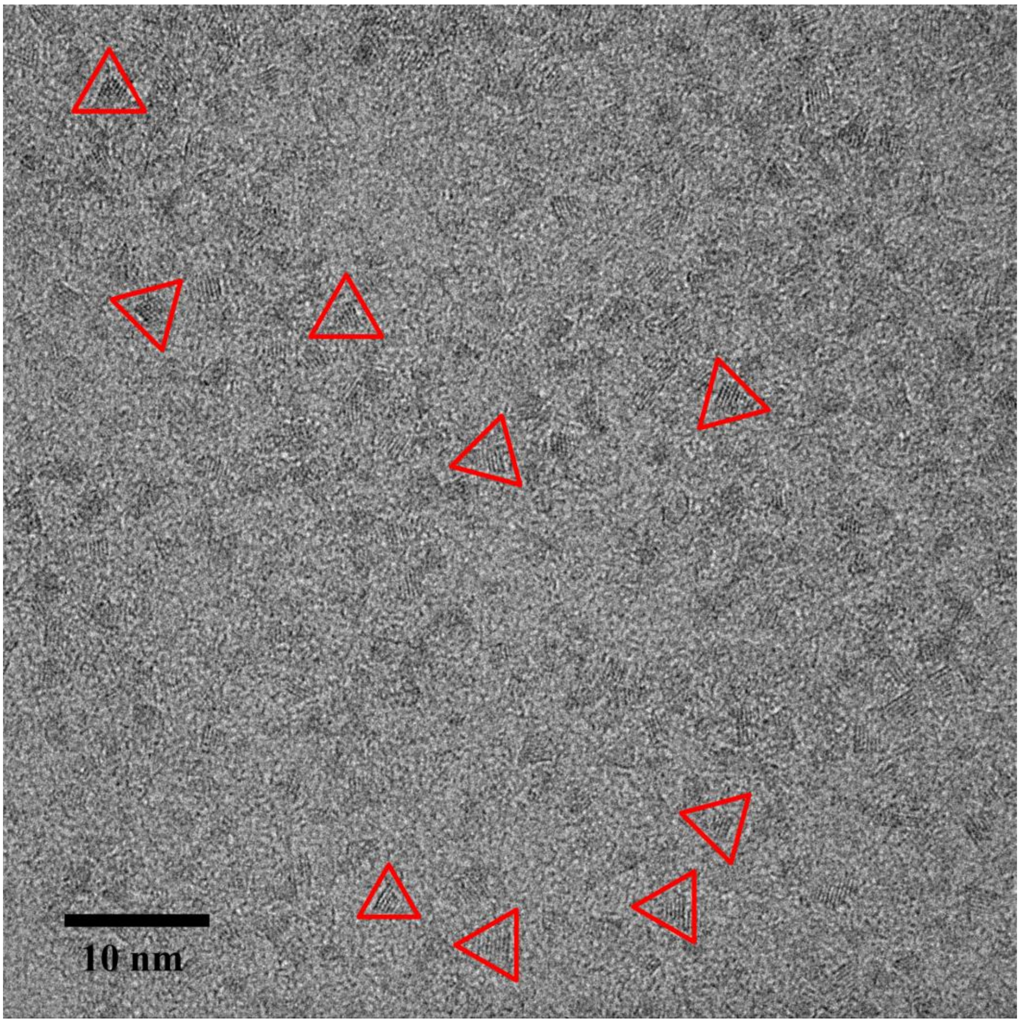

Supplementary Figure 21. A representative TEM image of (Zn)CuInS alloyed QDs (the stoichiometric ratio of Cu:In:Zn is 1:4:3) with etching time of 90 min. The red triangle frames indicate the shapes of the (Zn)CuInS alloyed QDs.

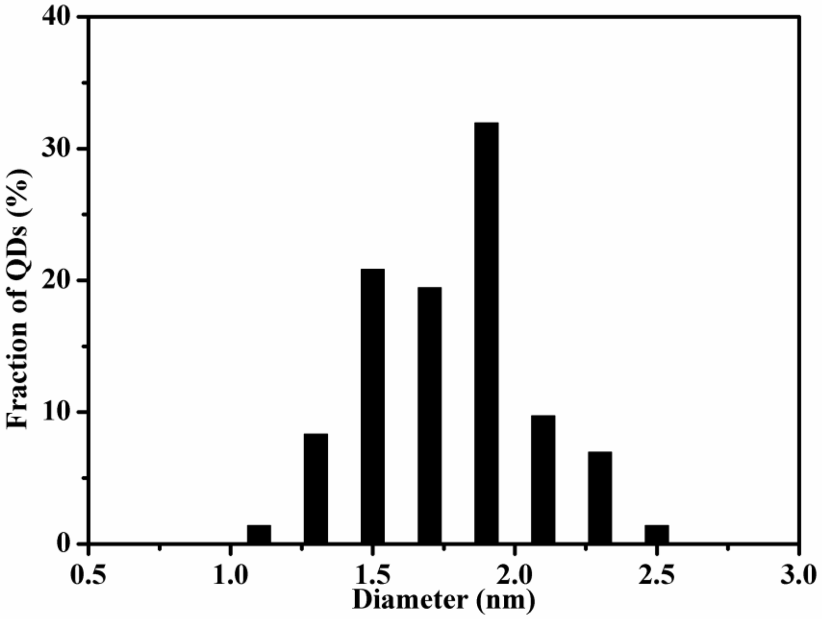

Supplementary Figure 22. Particle distribution histograms for (Zn)CuInS alloyed QDs (the stoichiometric ratio of Cu:In:Zn is 1:2:3) with etching time of 90 min. To build the histograms, the longest dimension for over 200 particles in each sample has been measured. The average diameter of QDs is about 1.7 nm.

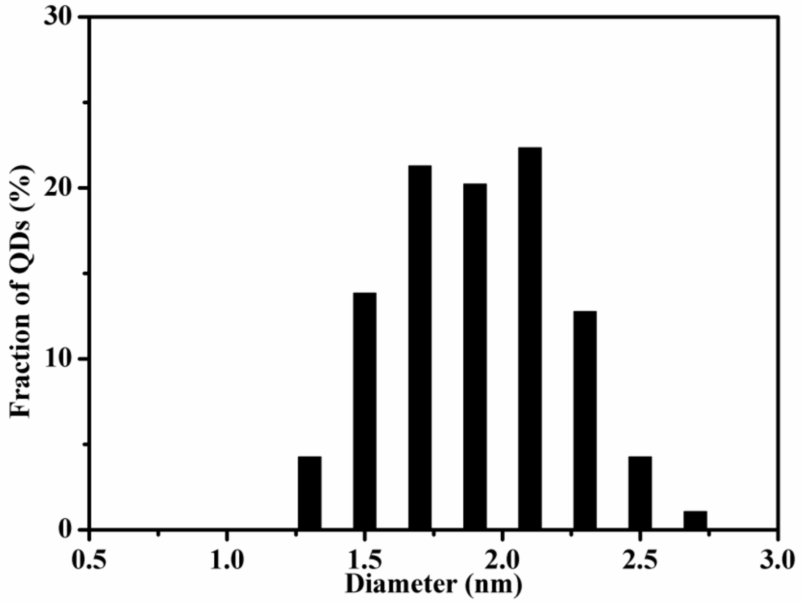

Supplementary Figure 23. Particle distribution histograms for (Zn)CuInS alloyed QDs (the stoichiometric ratio of Cu:In:Zn is 1:4:3) with etching time of 90 min. To build the histograms, the longest dimension for over 200 particles in each sample has been measured. The average diameter of QDs is about 1.9 nm.

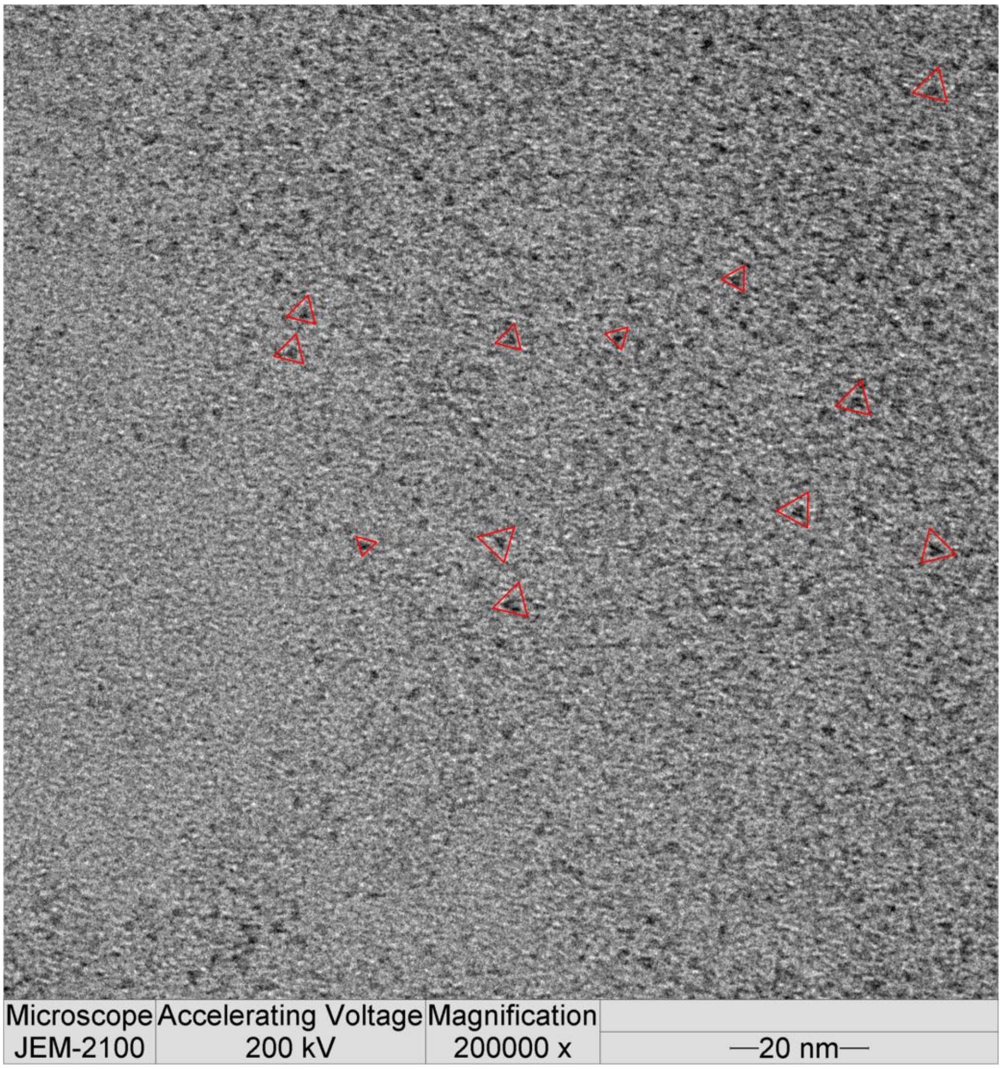

Supplementary Figure 24. A representative TEM image of (Zn)CuInS alloyed QDs (the stoichiometric ratio of Cu:In:Zn is 1:1:3) with etching time of 90 min. The red triangle frames indicate the shapes of the (Zn)CuInS alloyed QDs.

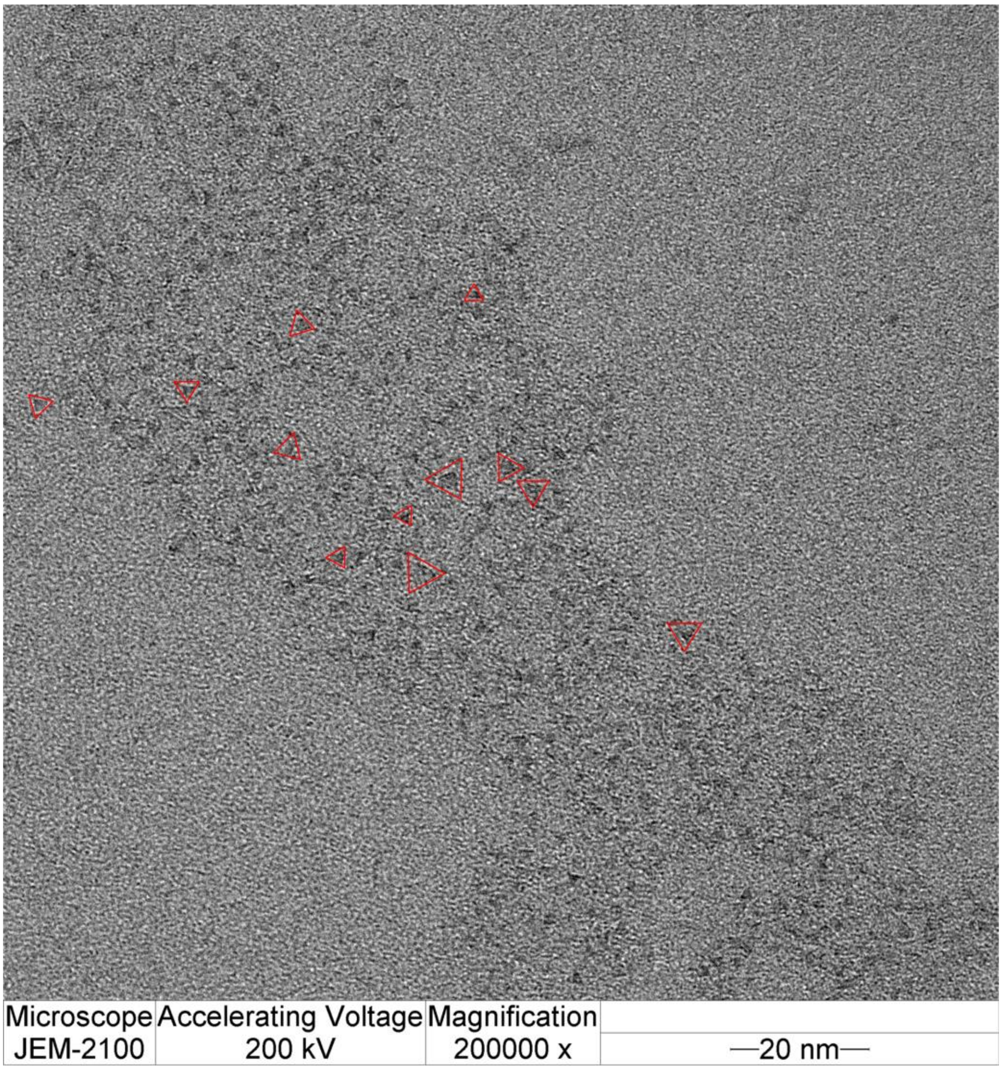

Supplementary Figure 25. A representative TEM image of (Zn)CuInS alloyed QDs (the stoichiometric ratio of Cu:In:Zn is 1:6:3) with etching time of 90 min. The red triangle frames indicate the shapes of the (Zn)CuInS alloyed QDs.

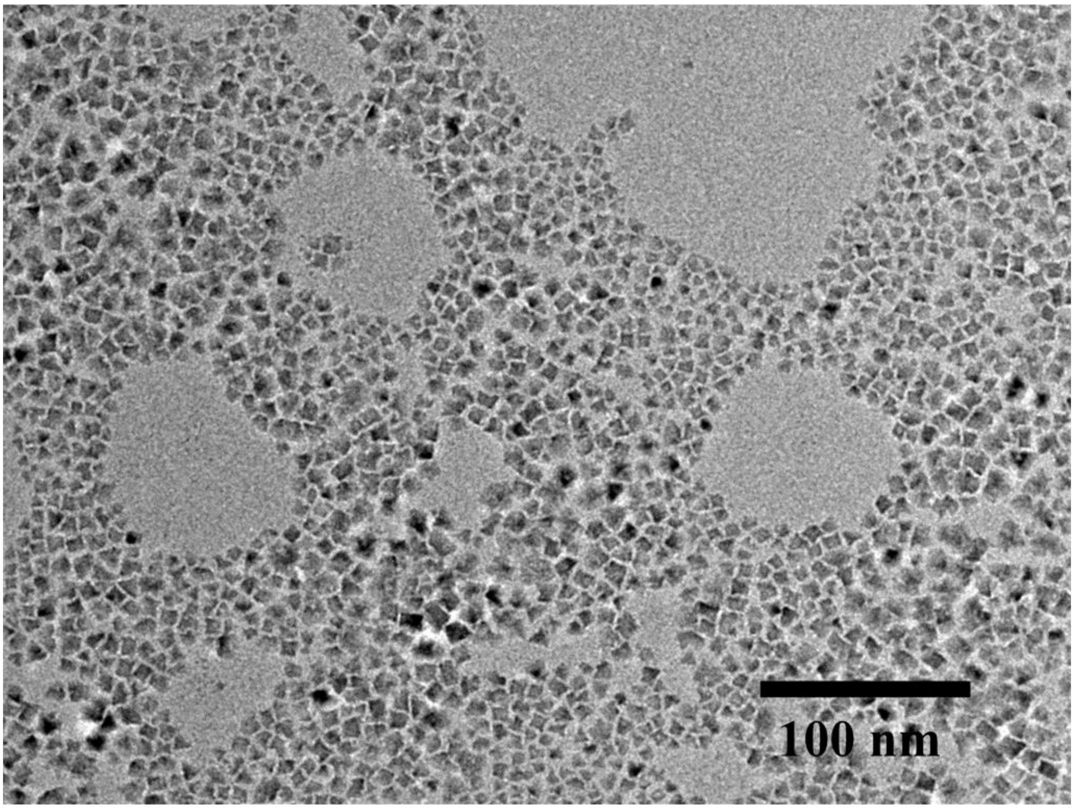

Supplementary Figure 26. A representative TEM image of Zn(CuInS)/ZnS QDs (the stoichiometric ratio of Cu:In:Zn is 1:1:3) with ZnS shell growth time of 20 h.

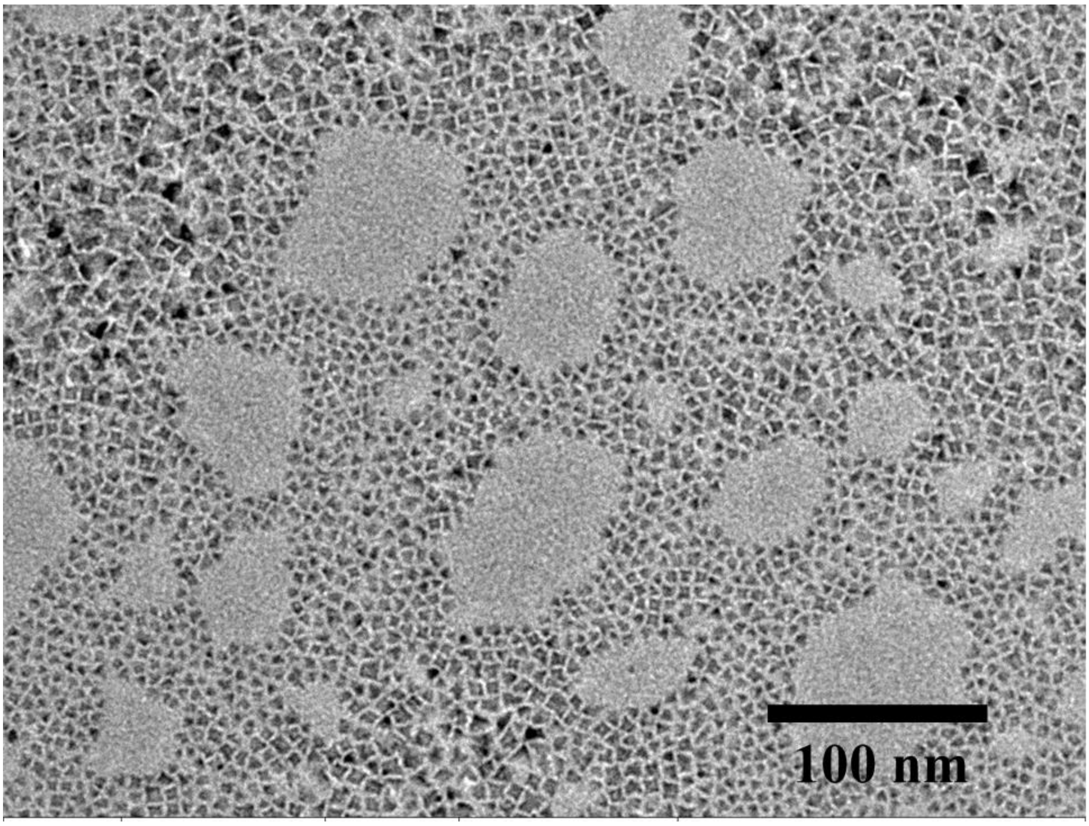

Supplementary Figure 27. A representative TEM image of Zn(CuInS)/ZnS QDs (the stoichiometric ratio of Cu:In:Zn is 1:6:3) with ZnS shell growth time of 20 h.

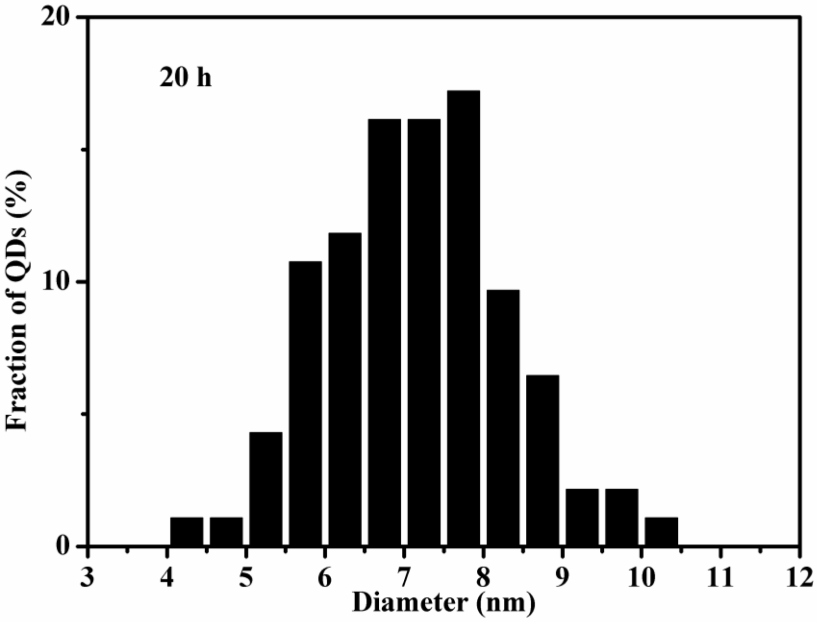

Supplementary Figure 28. Particle distribution histograms for (Zn)CuInS/ZnS QDs (the stoichiometric ratio of Cu:In:Zn is 1:1:3) with ZnS shell growth time of 20 h. To build the histograms, the longest dimension for over 200 particles in each sample has been measured. The average diameter of QDs is about 7.8 nm.

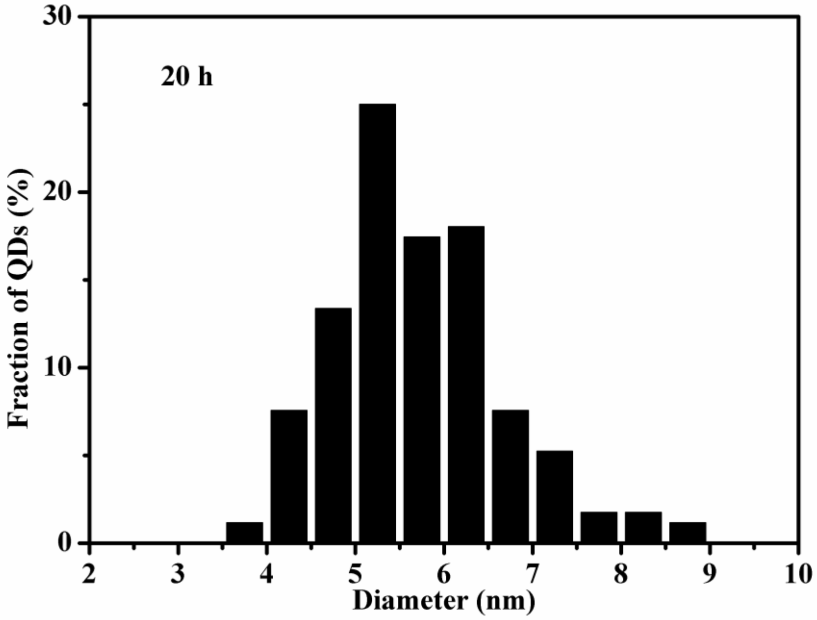

Supplementary Figure 29. Particle distribution histograms for (Zn)CuInS/ZnS QDs (the stoichiometric ratio of Cu:In:Zn is 1:6:3) with ZnS shell growth time of 20 h. To build the histograms, the longest dimension for over 200 particles in each sample has been measured. The average diameter of QDs is about 7.3 nm.

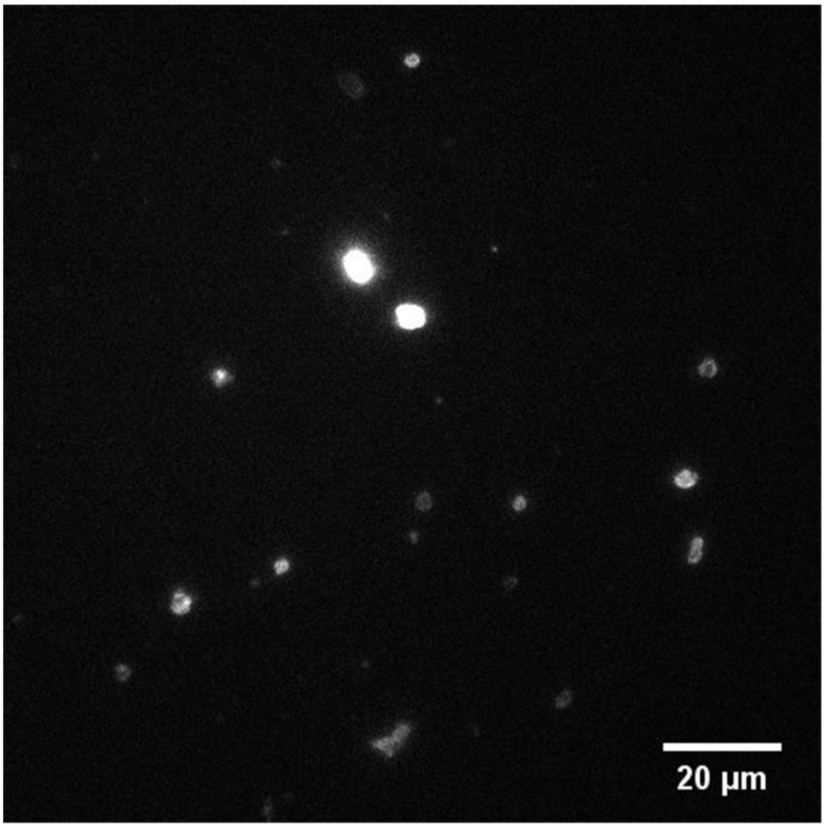

Supplementary Figure 30. A typical TIRFM images of (Zn)CuInS alloyed QDs (the stoichiometric ratio of Cu:In:Zn is 1:2:3) on glass substrate embedded in a PMMA (2%, g/mL) matrix at room temperature. QDs are seriously aggregated observed in this case. The exposure time is 50 ms.

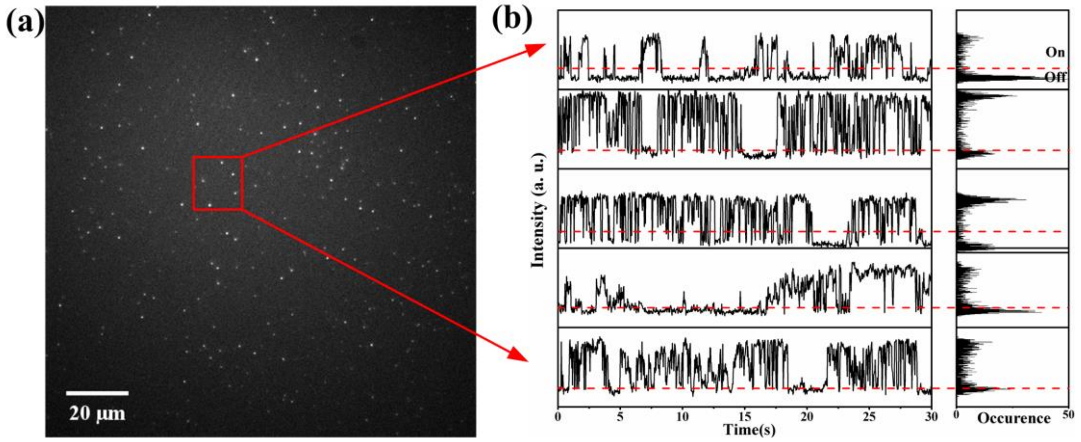

Supplementary Figure 31. (a) Typical TIRFM images of thiol-modified CdSe/CdS QDs on glass substrate dispersed with mixed organic solvent (90% hexane-10% octane) at room temperature. These QDs were prepared with 1-dodecanethiol as S precursors and surface ligand with particle size of about 5-6 nm. QDs are well separated and no aggregation is observed in this case. The exposure time: 30 ms. (b) Temporal evolution of representative PL intensity trajectories for five randomly selected thiol-modified CdSe/CdS QDs. Histograms indicate the corresponding distribution of intensities observed in the trajectories. The dashed red line is the chosen value as the threshold between “on time” and “off time”.

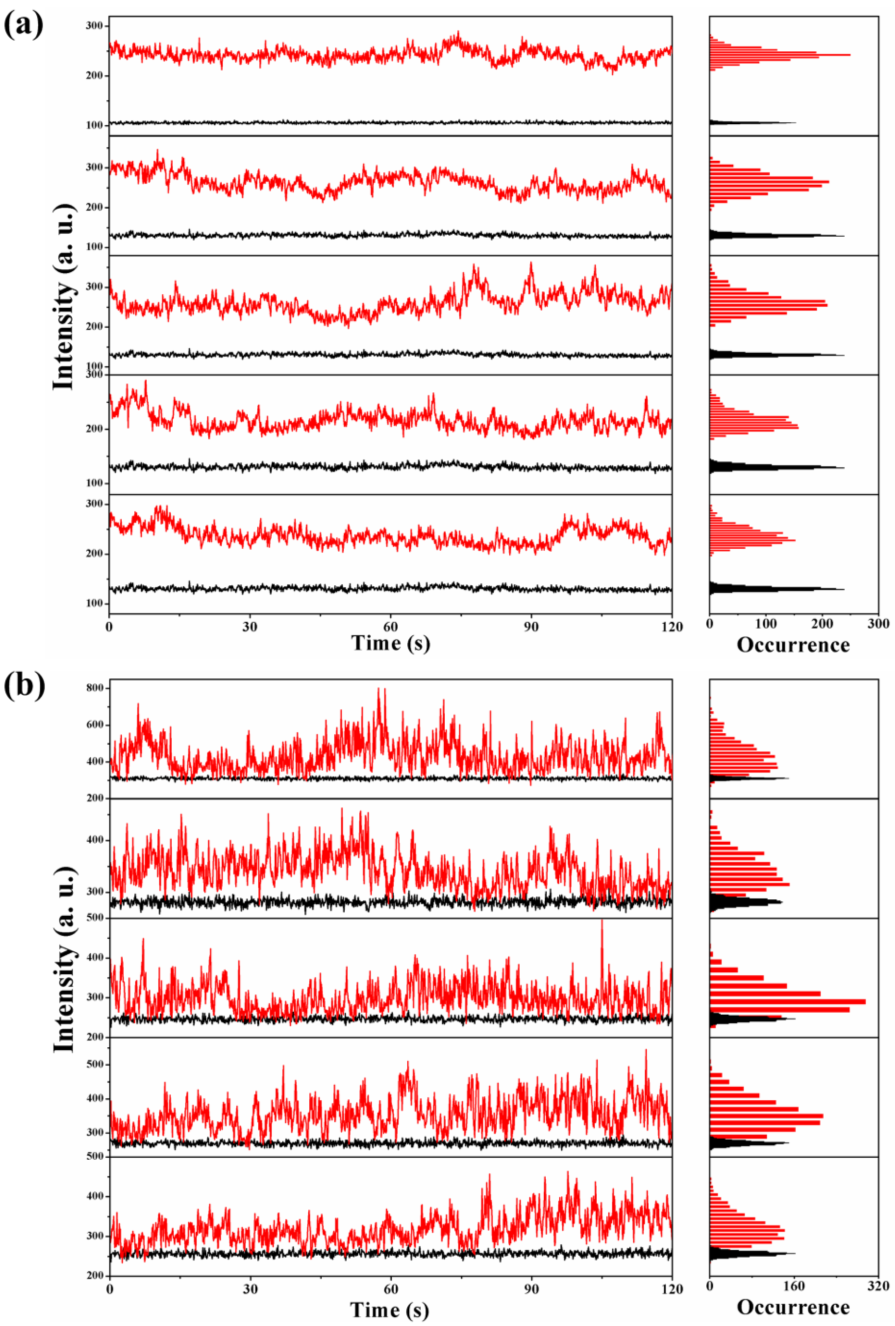

Supplementary Figure 32. (a) Temporal evolution of representative fluorescence-intensity trajectories for (Zn)CuInS/ZnS QDs with ZnS shell growth time of 20 h. (the stoichiometric ratio of Cu:In:Zn is 1:2:3). All QDs were excited with a 450 nm pulse laser. (b) Temporal evolution of representative fluorescence-intensity trajectories for (Zn)CuInS/ZnS QDs with ZnS shell growth time of 20 h. (the stoichiometric ratio of Cu:In:Zn is 1:2:3). All QDs were excited by a 405 nm continuous-wave laser. Histograms to right indicate the corresponding distribution of intensities observed in the trajectories. The data were recorded by an EMCCD (Andor DU-897) with offset correction. The binning time is 100 ms. The horizontal black line is the intensity of the background fluorescence signal.

Supplementary Table 1. Relevant parameters for CuInS QDs prepared with different stoichiometric ratios of Cu:In[a]

Cu:In precursor	Actual ratios of Cu:In:S	λ_{em} (nm)	QY (%)
1:1	1.00 : 1.09 : 3.16	710	3.3
1:2	1.00 : 1.20 : 3.20	633	8.8
1:4	1.00 : 1.97 : 3.04	625	18.9
1:6	1.00 : 2.51 : 3.53	618	21.0

[a]The composition of the CuInS QDs were measured by EDS and ICP-OES. λ_{em} is the wavelength corresponding to the maximum of the PL emission peak from the QDs solutions when excited at 450 nm. The amounts of DDT (10 mL) and CuI (0.1 mmol) were held fixed. The actual ratios of Cu:In:S are normalized to the molar quantity of the Cu element.

Supplementary Table 2. Relevant parameters for (Zn)CuInS alloyed QDs prepared with different stoichiometric ratios of Cu:In:Zn[a]

Cu:In precursor	Actual ratios of Cu:In:Zn	λ_{em} (nm)	QY (%)
1:1	1.00 : 4.58 : 1.63	675	14.7
1:2	1.00 : 3.95 : 2.33	596	16.8
1:4	1.00 : 4.51 : 1.93	590	24.8
1:6	1.00 : 3.35 : 1.57	581	36.3

[a]The composition of the (Zn)CuInS QDs were measured by EDS and ICP-OES. λ_{em} is the wavelength corresponding to the maximum of the PL emission peak from the QDs solutions when excited at 450 nm. The amounts of DDT (10 mL), CuI (0.1 mmol), and zinc stearate (0.3 mmol) were held fixed. The actual ratios of Cu:In:Zn are normalized to the molar quantity of the Cu element.

Supplementary Table 3. Relevant parameters for (Zn)CuInS/ZnS QDs with different ZnS shell growth time prepared with the (Zn)CuInS alloyed QDs (Cu:In:Zn stoichiometric ratio of 1:2:3)[a]

Shell growth time (h)	Actual ratios of Cu:In:Zn
5	1.00:1.41:30.96
10	1.00:1.31:33.05
15	1.00:1.28:34.83
20	1.00:1.37:36.97

[a]The composition of the (Zn)CuInS/ZnS QDs were measured by EDS and ICP-OES. The amounts of DDT (10 mL), CuI (0.1 mmol), $In(Ac)_3$ (0.2 mmol), and zinc stearate (0.3 mmol) for synthesizing (Zn)CuInS QDs were held fixed. The actual ratios of Cu:In:Zn are normalized to the molar quantity of the Cu element.

Supplementary Table 4. Relevant parameters for (Zn)CuInS/ZnS QDs with different ZnS shell growth time prepared with the (Zn)CuInS alloyed QDs (Cu:In:Zn stoichiometric ratio of 1:4:3)[a]

Shell growth time (h)	Actual ratios of Cu:In:Zn
5	1.00:2.85:35.16
10	1.00:2.35:44.64
15	1.00:2.01:43.17
20	1.00:1.13:48.52

[a]The composition of the (Zn)CuInS/ZnS QDs were measured by EDS and ICP-OES. The amounts of DDT (10 mL), CuI (0.1 mmol), $In(Ac)_3$ (0.4 mmol), and zinc stearate (0.3 mmol) for synthesizing (Zn)CuInS QDs were held fixed. The actual ratios of Cu:In:Zn are normalized to the molar quantity of the Cu element.

Supplementary References

1. De Trizio, L., *et al.* Strongly fluorescent quaternary Cu-In-Zn-S nanocrystals prepared from $Cu_{1-x}InS_2$ nanocrystals by partial cation exchange. *Chem. Mater.* **24,** 2400-2406 (2012).
2. Zan, F., Dong, C., Liu, H., Ren, J. Experimental studies on blinking behavior of single InP/ZnS quantum dots: effects of synthetic conditions and UV irradiation. *J. Phys. Chem. C* **116,** 3944-3950 (2012).
3. Zhang, A., Dong, C., Liu, H., Ren, J. Blinking behavior of CdSe/CdS quantum dots controlled by alkylthiols as surface trap modifiers. *J. Phys. Chem. C* **117,** 24592-24600 (2013).
4. Mahler, B., *et al.* Towards non-blinking colloidal quantum dots. *Nat. Mater.* **7,** 659-664 (2008).
5. Hanley, C. A., *et al.* Preparation and investigation of quantum-dot-loaded hollow polymer microspheres. *J. Phys. Chem. C* **117,** 24527-24536 (2013).
6. Ha, T. Photonics: How nanocrystals lost their blink. *Nature* **459,** 649-650 (2009).
7. Qin, H., *et al.* Single-dot spectroscopy of zinc-blende CdSe/CdS core/shell nanocrystals: nonblinking and correlation with ensemble measurements. *J. Am. Chem. Soc.* **136,** 179-187 (2013).
